# Cognitive Behavioural Therapy and Dual Diagnosis: A Systematic Review Exploring Its Effectiveness and Implications for Nursing Practice

**DOI:** 10.1111/inm.70129

**Published:** 2025-10-18

**Authors:** Dominic Nessbach, Alan Simpson

**Affiliations:** ^1^ Florence Nightingale Faculty of Nursing, Midwifery & Palliative Care James Clerk Maxwell Building, Kings College London Waterloo Campus London UK

**Keywords:** cognitive behavioural therapy, dual diagnosis, mental health, mental health nurse, substance use

## Abstract

Dual diagnosis (DD) is defined as the presence of a co‐occurring mental health and substance use disorder. It is associated with poor treatment outcomes, which can be further fuelled by frequent exclusion from specialist treatment due to the separation between mental health and drug and alcohol services. Cognitive Behavioural Therapy (CBT) has an extensive evidence base in treating mental health and substance use disorders in isolation, but there is a paucity of evidence regarding its efficacy in treating DD. The current systematic review aimed to explore the use and effectiveness of CBT as a treatment for individuals with DD. Sources were derived in September 2024 from electronic databases including Medline, PsychINFO, Embase and CINAHL; topically relevant meta‐analyses were also citation tracked. Twenty‐three studies were included in this review from a total of 2364 which were initially retrieved. Study outcomes highlighted that CBT‐based interventions provided some level of improvement to mental health or substance use symptoms, although several interventions did not display superiority when compared to typical addiction approaches. Mental health nurses are well suited to deliver CBT‐based interventions and could address the current treatment gap experienced by individuals with DD. This could include supporting patients in maintaining and generalising CBT skills that have already been acquired, which would help guarantee accessibility to CBT‐based interventions over a longer time period. However, additional support structures would need to be implemented to allow nurses to deliver CBT effectively, such as access to training, supervision, protected time and reflective practice.

## 1 Introduction

Dual diagnosis (DD) is the presence of a co‐occurring substance use problem and mental health condition (Hakobyan et al. 2020). In England, Public Health England (2017a) highlighted that in community drug and alcohol (DA) settings, MH difficulties are experienced by 70% and 86% of DA users respectively. In 2021, 63% of people receiving community DA treatment reported having a MH need (Department of Health and Social Care 2021). Furthermore, 75% of people with a severe MH condition (i.e., individuals with secondary MH service involvement) have a DD. Mood and anxiety disorders, personality disorders (primarily emotionally unstable and antisocial) and psychotic disorders have the highest co‐occurrence with substance use disorder (SUD) (Temmingh et al. 2018).

**FIGURE 1:**
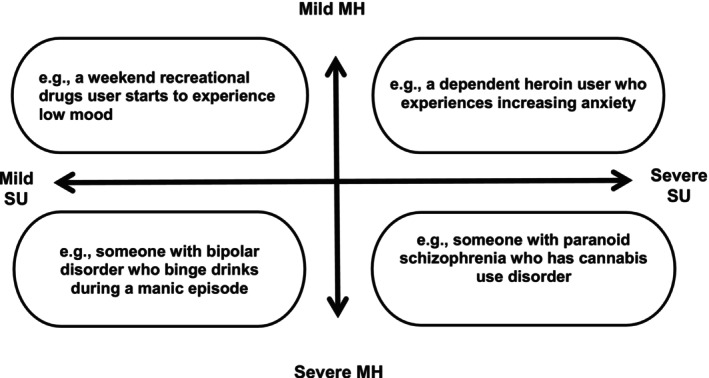
The scope of dual diagnosis, adapted from the Department of Health's dual diagnosis good practice guide (2002).

DD is heterogeneous in presentation, with patterns of consumption and presentations of mental distress that vary in severity. Therefore, it would be reductive to constrain the definition of DD to a set of characteristics. The UK Department of Health's (2002) quadrant best represents the variety of DD presentations (Figure 1). DD worsens treatment outcomes and prognoses within MH services, with a higher frequency of inpatient psychiatric admissions, a delayed remission of psychiatric symptoms and a greater likelihood of medication noncompliance and subsequent relapse (Fantuzzi and Mezzina 2020; Adan and Torrens 2021). These additional risks further complicate DD interventions. The aetiology of DD is multidirectional in nature. For example, substance use and withdrawal can lead to the emergence or exacerbation of MH symptoms for some, whilst others may use substances to alleviate pre‐existing MH symptoms. Such issues with disease chronology have contributed to diagnostic inaccuracies and fragmented service provision for those with DD (Pacini et al. 2020). Clinicians often lack the expertise required to identify and treat comorbidities involved in DD, increasing the likelihood of diagnostic overshadowing (Garrod et al. 2020; Gournay et al. 1997). Therefore, people with DD pose a unique challenge to healthcare services due to the multiplicity of their difficulties.

In the United Kingdom, treatment provision lacks specific and adapted interventions which address the severity, complexity and diversity of needs of individuals with DD. The consensus is that treatment should be delivered using an integrated care model, where substance use and MH are addressed within the same setting. Karapareddy (2019) found that integrated care models were superior to parallel and sequential treatment models in terms of positive treatment outcomes and cost effectiveness. The Department of Health (2002) advocated for the ‘mainstreaming’ of DD care, advising that MH clinicians should integrate substance use and MH into routine care and treatment planning. More recently, the Department of Health's (2017) ‘*Orange Guidelines*’ stated that interventions for both MH and substance use should be available in all services working with DD. The National Institute for Health and Care Excellence (2016) also highlighted the need for service collaboration to cater to the specific and unmet needs of patients with DD, meaning that all services should be anti‐discriminatory and proactive in offering assessments, referrals and treatment (Department of Health 2017).

Despite this policy direction, MH and DA services are designed, commissioned and provided in almost complete separation. This is also the case for other countries including Canada, Australia and the United States, and is a significant barrier to continuity of care for individuals with DD (Glover‐Wright et al. 2023). In 2013, DA services in the United Kingdom had their protected funding source withdrawn and replaced by the public health grant (The King's Fund 2020). This is a lump sum paid to local authorities by the Department of Health (now Department of Health and Social Care) which is divided across all preventative health services, including DA services. This has resulted in a 28% reduction in funding between 2015/16 and 2022/23, leaving services with less autonomy and scope to develop and an increasing inability to adapt service provision to include people with co‐occurring MH issues (The Kings Fund 2020).

MH services, particularly community MH and crisis teams, maintain that they cannot support people presenting with distress through substance use issues (Department of Health 2017). Primary MH services, such as NHS Talking Therapies, that provide evidence‐based psychological treatments, primarily cognitive behavioural therapy (CBT) for depression and anxiety disorders, are often the first point of call for a person experiencing MH difficulties. They regularly exclude people if they use substances or are unable to maintain abstinence for several months following DA treatment, contrary to positive practice guidelines (The National Collaborating Centre for Mental Health 2018). Shahriyarmolki et al. (2025) found that clinicians' rationale for rejecting DA clients often reflected misconceptions, including that substance use would render therapy ineffective and that treatment would exacerbate DA use. MH services also rarely provide CBT interventions that target substance use for individuals who are accepted into treatment, despite an extensive evidence base that CBT is an effective treatment for SUD (McHugh et al. 2010).

### 1.1 Cognitive Behavioural Therapy and Dual Diagnosis

**FIGURE 2:**
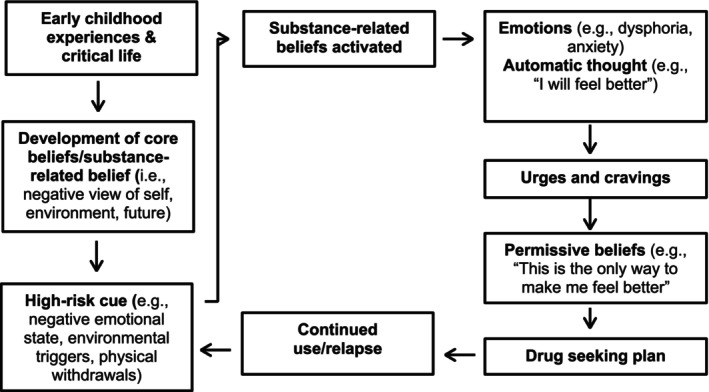
Cognitive therapy model of substance abuse adapted from Beck et al. (1993).

CBT is a structured psychotherapeutic approach which posits that implicit thinking biases are fundamental in the development and maintenance of maladaptive behaviours which contribute to an individual's mental distress (Fenn and Byrne 2013). By utilising techniques to make these thoughts explicit, and equipping people with tools to develop alternative thoughts, dysfunctional patterns of cognition and behaviour can be changed (Mitcheson et al. 2010). Beck et al. (1993), amongst others, have developed disease‐specific models of CBT which have been used to inform substance use interventions. In this model (Figure 2), core beliefs (or schemas), which include substance‐related beliefs that manifest due to past life experiences, are activated by high‐risk cues of internal or external origin. These beliefs give rise to emotions and automatic substance‐related thoughts, such as anticipatory beliefs pertaining to the perceived reward of taking a substance, which result in urges and cravings. As individuals start to associate substance use with a relief of mental distress, they develop permissive and relief‐orientated beliefs which, once activated, result in drug‐seeking behaviours. Within this framework, the main aim of treatment is targeting core beliefs which underlie addictive behaviour (Newman 2019).

McHugh et al. (2010) conducted a meta‐analytic review of CBT for drug use and dependence including 34 randomised controlled trials (RCT) (with 2340 patients treated) and found an overall effect size in the moderate range (*d* = 0.45), with effect sizes ranging from small (*d* = 0.24) to large (*d* = 0.81) depending on the substance targeted. They found a limited number of studies investigating effectiveness in routine MH services. Magill and Ray (2009) also reported similar rates of efficacy for CBT in people with alcohol use disorder (AUD). CBT is also efficacious in treating a variety of MH symptoms experienced by individuals with DD. Hofmann et al. (2012) reviewed a sample of 106 meta‐analytic reviews of CBT for MH conditions and found medium to large effect sizes for depression and anxiety (similar findings also found in Carpenter et al.'s (2018) meta‐analysis of 41 RCT's with 2843 patients); a small to medium effect size for bipolar disorder and a medium effect size for positive and negative symptoms of schizophrenia (specific effect size values not provided).

**FIGURE 3:**
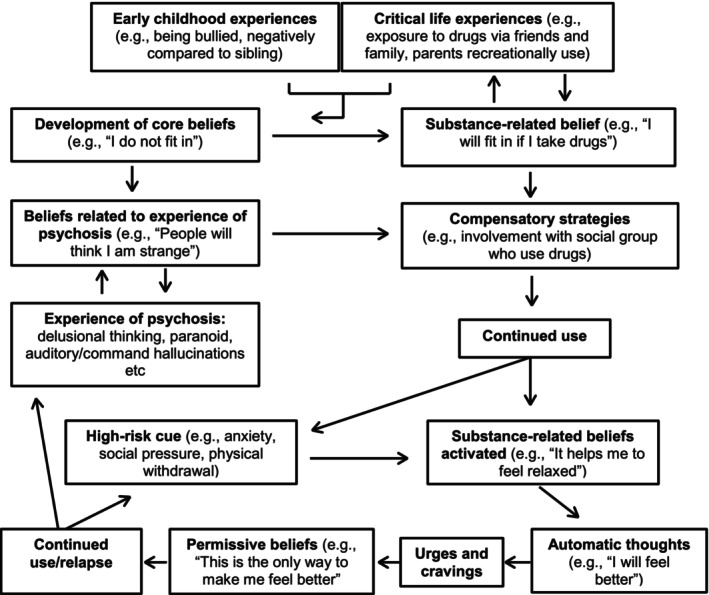
Cognitive model of a person with comorbid substance use and psychosis.

Cognitive‐behavioural integrated treatment (C‐BIT), developed by Graham (2003), is a treatment approach which applies the cognitive model of substance use to those with severe MH problems. It focuses on how substance‐related beliefs are linked to an individual's experience of MH, including how beliefs function to support an individual to manage their MH (Figure 3). C‐BIT uses motivational, social and behavioural elements (e.g., motivational interviewing) in conjunction with CBT techniques to engender a shift in an individual's core beliefs. This is a prime example of how DD treatment could be implemented using an integrated care model. However, this is rarely practiced due to the disconnection between MH and DA services and a lack of clinicians with sufficient skills and knowledge in these approaches (Petrakis et al. 2018).

Nurses have been working with DD patients for several decades, both within DA services and other settings as roles have diversified. According to Public Health England (2017b), the nursing workforce within DA settings historically consisted of mental health nurses (MHN). The role and responsibilities of MHNs working with DD have expanded to include prescribing and, in some areas, specialist practitioner roles. Nurses are well equipped to deliver psychosocial interventions for co‐occurring MH conditions such as anxiety and depression (e.g., coping strategy enhancement) within DA and MH settings (Public Health England 2017b). In addition, the Nursing and Midwifery Council (2018) highlights that practice must be evidence based, requiring clinicians to deliver the most effective form of treatment available. Considering there is evidence to support the use of CBT within substance use settings, MHNs should already be delivering CBT‐informed practice. The National Collaborating Centre for Mental Health (2018) posits that CBT techniques used by therapists are easily transferrable to those working with DD. Sims (2022) suggested that there could be scope for an enhanced nursing role where MHNs working in substance use are trained to deliver CBT under the supervision of a colleague accredited in CBT therapy. Therefore, nurses are well positioned to deliver CBT‐based interventions to people with DD who are unable to access such treatment.

## 2 Aims

The aim of this review is to investigate the effectiveness of CBT in the treatment of MH and substance use within the DD population. Secondary aims were to explore:

- The effectiveness of CBT as a monotherapy and as part of combined treatment strategies, with consideration of specific aspects of comorbidity (i.e., type of SUD, severity of dependence, and MH condition).
- To provide recommendations on how CBT‐based practice can be better integrated into nursing practice.

## 3 Methods

A systematic review was conducted in accordance with the Preferred Reporting Items for Systematic Reviews and Meta‐Analyses (PRISMA) guidelines (Page et al. 2021). The PRISMA checklist can be found in Table S1. Unfortunately, we did not pre‐register the study as the initial work was undertaken as part of a university degree dissertation.

### 3.1 Search Strategy

The following databases were searched for relevant papers: CINAHL, PubMed (Medline), Embase and PsychInfo. The population, context and outcome (PCO) strategy was used to identify and select relevant studies. A title, abstract and keyword search was conducted (in September 2024) in the PubMed (Medline) database using the following search terms: (Diagnosis, Dual (Psychiatry)) OR (Organic Mental Disorders, Substance Induced) AND (Therapy, Cognitive Behavioural) OR (Therapy, Cognitive Behavioural) OR (Cognitive Behavioural Therapy) AND outcome (‘alcohol’ OR ‘cocaine’ OR ‘stimulant’ OR ‘opiate’ OR ‘heroin’ OR ‘marijuana’ OR ‘cannabis’ OR ‘substances’ OR ‘polysubstance’ OR ‘dual disorder’ OR ‘dual diagnosis’ OR ‘co‐occurring disorder’ OR ‘comorbid disorder’). CINAHL, Embase and PsychINFO were then accessed, excluding any records from Medline—see Table S2a,b for the search stream applied to all databases, including filters and limits applied to each search. Due to time and resource constraints, we did not conduct a search of grey literature. Once the search had been completed, titles of studies were screened for relevance. Abstracts were then screened to assess whether they had met eligibility criteria and were then accessed in full text to assess for inclusion during the final stage of screening. Topically relevant systematic reviews and meta‐analyses were also citation tracked in order to identify additional studies that had been missed by the original search (Mehta et al. 2021; Roberts et al. 2022); reference lists of selected studies were also searched. The selection process was performed by the first author only as part of a university degree dissertation; the second author provided supervision but was unable to work on data extraction.

### 3.2 Inclusion and Exclusion Criteria

Empirical papers published after 2010 were included in order to focus on the most recent and relevant literature to current practice. Additional inclusion criteria included: (1) adult sample (as focus was on adult community MH services); (2) participants with a DD presentation (i.e., SUD and diagnosed MH condition); (3) sample must have received CBT or CBT‐based treatment which explicitly targeted both MH and SUD or explored the relationship between MH symptoms and substance use; (4) inclusion of MH and substance use outcome measures. Exclusion criteria included: (1) study protocols; (2) sample with SUD only; (3) use of third‐wave CBT (e.g., Acceptance and Commitment Therapy) or self‐help interventions; (4) sample where co‐occurring condition was of neurodevelopmental origin (e.g., ADHD); (5) non‐English language.

### 3.3 Quality Appraisal

The Revised Cochrane Risk of Bias tool (RoB 2) was used to appraise the quality of RCTs in this review (Higgins et al. 2019). The RoB 2 assesses bias in five domains: the randomisation process, deviations from intended interventions, missing outcome data, measurement of the outcome and the selection of reported results. Each domain has signalling questions with response guidance, which culminate in an overall risk of bias judgement that is calculated by an algorithm. Studies were rated as either at low risk, some concerns or high risk of bias. As the primary aim of this review was to assess the effectiveness of CBT interventions in treating DD, the effect of assignment to intervention was focused on (i.e., intention‐to‐treat (ITT) analysis).

### 3.4 Data Extraction and Synthesis

Clinical trial data was synthesised from CBT interventions through the extraction of key study characteristics from the selected studies, including sample size and aetiology, demographic information (i.e., age and gender), a brief description of the target and comparator intervention and the main findings obtained from SUD and MH outcome measures. Means, standard deviations, 95% confidence intervals and effect sizes were extracted for all outcome measures, and if unavailable, they were calculated where possible. Due to the methodological and clinical heterogeneity present across the selected studies, it was not possible to perform a meta‐analysis.

## 4 Results

### 4.1 Search Outcomes

**FIGURE 4:**
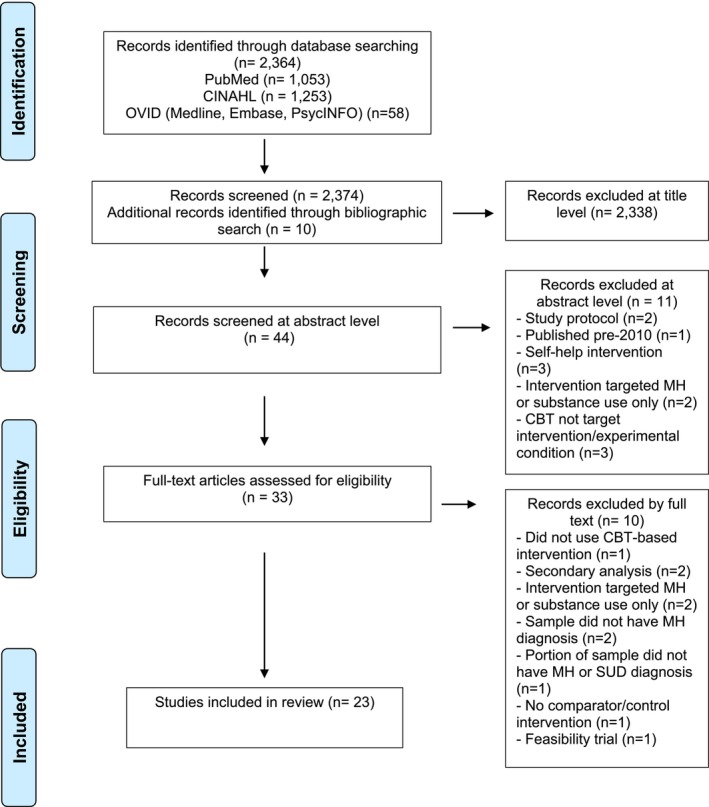
Preferred Reporting Items for Systematic Reviews and Meta‐Analyses (PRISMA) flow diagram.

A total of 2364 papers were identified following the removal of duplicates, with an additional 10 papers being identified through bibliographic searching. In this study, 2338 papers were excluded at the title level as they did not include key words that reflected the search terms, nor fit the eligibility criteria. Forty‐four papers were screened at the abstract level, with 11 being excluded at this stage due to the type of intervention (i.e., self help interventions, not targeting mental health and substance use) or study (i.e., protocol) not meeting inclusion criteria. Thirty‐three papers were fully accessed for eligibility screening. Twenty‐four papers were selected for inclusion in the current project; a visual representation of the study selection process can be seen in Figure 4. Table 1 shows the selected studies from the search; mean outcome data and standard deviations were available for extraction in most cases (with the exception of Hunter et al. 2012).

**TABLE 1:** CBT interventions for dual diagnosis.

Author, location	Participants, SUD type, age (mean), gender (male/female)	Target/comparator interventions	Outcome measures	Main findings: mean (M), standard deviation (SD), confidence intervals (95% CI), effect size (*d*\OR/IRR)^1^
Back et al. (2019) USA	Treatment seeking military veterans with PTSD diagnosis (*n* = 81) **SUD**: Alcohol only (*n* = 51), drug only (*n* = 8), alcohol and drug (*n* = 22) **Age**: 40.4 **Gender**: 90.1% M, 8.9% F	**Target**: Concurrent treatment of PTSD and Substance Use Disorders using Prolonged Exposure (COPE) (*n* = 54) – *Psychoeducation and forming connections beteen PTSD and substance use*, in vivo *and imaginal exposure exercises, coping with cravings* **Comparator**: Relapse Prevention (RP) (*n* = 27)	**MH**: CAPS, PCL‐M, BDI‐II **SUD**: TLFB, ASI **Measurement points**: baseline, weeks 6 and 12, 3 and 6‐month follow‐up	**PTSD symptom severity improved significantly more for participants receiving COPE on both outcome measures** ^**a**^ – *PCL‐M*: COPE (MD = −22.3, SD = 26.24, CI −29.3 to −15.3); RP (MD = −10.9, SD = 29.02, CI −18.0 to 3.9) – *CAPS*: COPE (MD = −51.2, SD = 31.68, CI −59.7 to −42.8); RP (MD = −35.9, SD = 33.00, CI −48.8 to −23.0) **Higher proportion of participants receiving COPE no longer met crtieria for PTSD** (59.3% [COPE] vs. 22.2% [RPT]; OR 5.3, CI 1.8–15.7)^b^ **Substance use decreased significantly in both groups, greater proportion of COPE participants reported abstinence during last 2 weeks of treatment** (42.6% [COPE] vs. 25.9% [RPT]) **Reductions in substance use maintained in both groups at follow‐up**
Barrowclough et al. (2010) UK	Outpatients with schizophrenia, schizophreniform or schizoaffective disorder (*n* = 327) **SUD**: Alcohol only (*n* = 157), drug only (*n* = 116), alcohol and drug (*n* = 54) **Age**: 37.8 **Gender**: 86.5% M, 13.5% F	**Target**: Integrated MI + CBT plus TAU (*n* = 164) – *Motivational building for substace use, exploring perspectives on substance use and MH goals, CBT techniques for substance use and psychosis. Up to 26 sessions* **Comparator**: TAU (*n* = 163)	**MH**: admission frequency/duration, relape frequency/duration, PANSS, GAF **SUD**: RCQ, InDUC, TLFB **Measurement points**: baseline, 6‐, 12‐, 18‐ and 24‐month follow up	**Higher proportion of MI + CBT group were admitted during follow‐up, but difference non‐significant** (22.1% [MI + CBT] vs. 17.2% [TAU], OR 1.16; CI 0.68–1.99) **No significant difference between groups on relapse in psychotic symptoms** (39.1% [MI + CBT] vs. 37.9% [TAU], OR 1.05, CI 0.67–1.66) **No difference between groups in frequency of substance use, but MI + CBT group found to consume less per day** (OR 1.48; 95% CI 1.07–2.05)^**b**^ **MI + CBT showed an increased readiness to change their substance use by month 12** (OR 2.05; 95% CI 1.26–3.31)^**b**^, **this was not maintained at 24 months**
Barrowclough et al. (2014) UK	Outpatients who have experienced first episode of psychosis (*n* = 110) **SUD**: Cannabis use/dependence (*n* = 110) **Age**: 24.1 **Gender**: 89% M, 11% F	**Target**: Integrated MI + CBT (*n* = 75) – Brief (12 sessions) (*n* = 38), long (24 sessions) (*n* = 37) – *Same as* *Barrowclough et al.* (2010) **Comparator**: TAU (*n* = 35)	**MH**: PANSS, GAF, BAI, CDSS **SUD**: RCQ, TLFB **Measurement points**: baseline, 4.5‐, 9‐ and 18‐month follow up	**No significant changes in cannabis use across all three treatment groups, no significant group by time interactions with regard to change in cannabis use frequency and amount. Small reduction in use (over 30 day period) in those receiving brief MI + CBT and TAU at 18**‐**month follow up** (Brief: M = −3.96 g, SD = 43.1; TAU: M = −4.33 g, SD = 31.4; Long: M = +9.25, SD = 45.1) **No significant differences between all three treatment groups in terms of psychotic symptom severity, global functioning and frequency of hospital admissions**
Buckner et al. (2019) USA	Participants who fit DSM‐IV criteria for anxiety disorder (*n* = 55) **SUD**: Cannabis use/dependence (*n* = 55) **Age**: 23.2 **Gender**: 56% M, 44% F	**Target**: Integrated Cannabis and Anxiety Reduction Treatment (ICART) (*n* = 27) – *Teaching ways of reducing false safety behaviours that maintain anxiety (*i.e., *cannabis use) in combination with skills to manage cravings in high‐risk situations, 12 sessions* **Comparator**: Motivational Enhancement Therapy‐CBT (MET + CBT) (*n* = 28)	**MH**: SIGH‐A **SUD**: TLFB, MPS, urine drug screen **Measurement points**: baseline, week 6, week 12	**Greater rates of abstinence in ICART compared to MET‐CBT by end of intervention (12%), but difference non‐significant** **Reduction in cannabis problem severity and self‐reported cannabis use in both interventions, group by‐time interactions not reliable** – *Problems*: ICART baseline (M = 15.18, SD = 6.74, CI 12.63–17.72), week 12 (M = 5.47, SD = 4.91, CI 3.61–7.32); MET‐CBT baseline (M = 10.67, SD = 6.15, CI 8.39–12.94), week 12 (M = 5.11, SD = 6.61, CI 2.66–7.55) (*d* = 0.06) – *Use*: ICART baseline (M = 60.47, SD = 37.16, CI), week 12 (M = 11.12, SD = 25.85), MET‐CBT baseline (M = 67.94, SD = 65.49, CI 43.68–92.19), week 12 (M = 22.89, SD = 43.56, CI 6.75–39.02) (*d* = 0.33) **Both treatments reduced anxiety, ICART superior** (*d* = 0.23) – *ICART*: baseline (M = 23.71, SD = 7.76, CI 20.78–26.63) – week 12 (M = 12.24, SD = 7.21, CI 9.56–14.91); MD = 11.47 – *MET‐CBT*: baseline (M = 17.83, SD = 7.20, CI 15.11–20.54) – week 12 (M = 10.61, SD = 6.91, CI 8.00–13.21); MD = 7.22
Capone et al. (2018) USA	Military veterans with PTSD diagnosis (*n* = 44) **SUD**: Alcohol (*n* = 37) and polydrug use—cannabis (*n* = 10), cocaine (*n* = 6), opioids (*n* = 3), sedatives (*n* = 1) **Age**: 34.3 **Gender**: 95% M, 5% F	**Target**: Integrated CBT (ICBT) (*n* = 21) – *8 modules: goal setting, relapse prevention, coping skills, psychoeducation on PTSD, cognitive restructuring, generalisation training, 8–12 sessions* **Comparator**: TAU (*n* = 23)	**MH**: CAPS **SUD**: TLFB, ASI **Measurement points**: baseline, post‐treatment, 3‐month follow up	**Significant time effects for PTSD symptom severity across both conditions for total score** ^**c**^, **re‐experiencing** ^**a**^, **avoidance/numbing** ^**b**^ **and hyperarousal** ^**b**^, **ICBT did not display superiority over TAU** – *Total*: ICBT baseline (M = 76.33, SD = 14.71, CI 70.03–82.62), 3 months (M = 66.07, SD = 17.60, CI 58.54–73.59); TAU baseline (M = 78.78, SD = 16.62, CI 71.98–85.57), 3 months (M = 62.27, SD = 26.26, CI 51.53–73.00) (*d* = 0.17) – *Re‐experiencing*: ICBT baseline (M = 23.29, SD = 5.66, CI 20.86–25.71), 3 months (M = 16.07, SD = 7.89, CI 12.69–19.44); TAU baseline (M = 21.48, SD = 7.67, CI 18.34–24.61), 3 months (M = 16.47, SD = 10.61, CI 12.13–20.80) (*d* = −0.04) – *Avoidance*: ICBT baseline (M = 29.24, SD = 7.67, CI 25.96–32.52), 3 months (M = 28.73, SD = 9.01, CI 24.87–32.58); TAU baseline (M = 32.57, SD = 6.68, CI 29.84–35.430), 3 months (M = 26.74, SD = 16.01, CI 20.19–33.28) (*d* = 0.15) – *Hyperarousal*: ICBT baseline (M = 23.81, SD = 5.02, CI 21.66–25.95), 3 months (M = 21.27, SD = 6.54, CI 18.47–24.06); TAU baseline (M = 24.74, SD = 6.13, CI 22.23–27.24), 3 months (M = 19.73, SD = 9.48, CI 15.85–23.60) (*d* = 0.99) **Significant reductions in alcohol and drug use severity over time and an increase in proportion of abstinent days** ^**a**^ **across both conditions, ICBT did not display superiority over TAU** – *Alcohol severity*: ICBT baseline (M = 0.31, SD = 0.22, CI 0.21–0.40), 3 months (M = 0.34, SD = 0.25, CI 0.23–0.44), TAU baseline (M = 0.41, SD = 0.28, CI 0.29–0.52), 3 months (M = 0.28, SD = 0.23, CI 0.18–0.37)^**c**^ (*d* = 0.25) – *Drug severity*: ICBT baseline (M = 0.12, SD = 0.16, CI 0.05–0.18), 3 months (M = 0.09, SD = 0.11, CI 0.04–0.13), TAU baseline (M = 0.10, SD = 0.11, CI 0.05–0.14), 3 months (M = 0.06, SD = 0.09, CI 0.02–0.09)^**b**^ (*d* = 0.30)
Coffey et al. (2016)	Inpatients of SUD treatment facility with PTSD diagnosis (*n* = 126) **SUD**: Alcohol only (*n* = 2), alcohol and polydrug use (*n* = 124) **Age**: 34 **Gender**: 54% M, 46% F	**Target**: Modified Prolonged Exposure, (mPE) (*n* = 45), mPE + MET‐PTSD (*n* = 40) – In vivo *and imaginal exposure, breathing retraining to manage PTSD‐associated arousal, education around relationship between PTSD and SUD, 9–12 sessions* **Comparator**: Healthy Lifestyles Sessions (HLS) (*n* = 41)	**MH**: IES‐R **SUD**: ACQ‐Now, ADS, TLFB (% days abstinent), urine drug screen **Measurement points**: baseline, post‐treatment, 6‐and 3‐month follow up	**mPE and mPE + MET‐PTSD conditions showed significantly greater reductions in PTSD symptoms compared to comparator but no significant difference between either target intervention. Reductions maintained at 3‐ and 6‐month follow up** – *mPE*: baseline (M = 48.6, SD = 17.54, CI 43.43–53.68), post‐treatment (M = 16.20, SD = 19.57, CI 10.48–21.92)^**b**^ – *mPE ± MET‐PTSD*: baseline (M = 54.95, SD = 17.55 CI 49.51–60.39), post‐treatment (M = 20.49, SD = 18.58, CI 14.77–26.20)^**a**^ **All conditions resulted in a significant reduction in alcohol and drug consumption** ^**c**^, **no significant group by time interaction** – *mPe*: baseline (M = 46.13, SD = 23.22, CI 39.04–52.61), 6 months (M = 94.49, SD = 22.43, CI 87.94–101.05) – *mPE ± MET‐PTSD*: baseline (M = 48.70, SD = 22.18, CI 41.82–55.57), 6 months (M = 85.73, SD = 26.75, CI 78.94–95.52) – *HLS*: baseline (M = 52.23, SD = 22.05, CI 45.53–59.03), 6 months (M = 93.58, SD = 22.19, CI 86.78–100.37) **All conditions resulted in a significant reduction in drug consumption** ^**c**^, **no significant group by time interaction** – *mPe*: baseline (M = 53.44, 95% CI 46.87–60.01), 6 months (M = 96.94, 95% CI 90.87–104.16) – *mPe ± MET‐PTSD*: baseline (M = 45.47, SD = 22.47, CI 38.50–52.43), 6 months (M = 91.97, SD = 22.21, CI 85.08–98.85) – *HLS*: baseline (M = 59.59, SD = 22.49, CI 52.71–66.48), 6 months (M = 91.70, SD = 22.49, CI 84.82–98.59) **All treatment conditions resulted in significant reductions in alcohol craving over the treatment course** ^**c**^, **no significant differences between target and comparator interventions**
Foa et al. (2013) USA	Treatment seeking participants with PTSD diagnosis (*n* = 165) **SUD**: Alcohol only (*n* = 165) **Age**: 42.7 **Gender**: 65.5% M, 34.5% F	**Target**: Exposure Therapy (ET) plus naltrexone (*n* = 40) – *Systematic confrontation (*via in vivo *or imaginal exposure) of feared internal or external stimuli using a graded approach, can be paired with relaxation exercises or interoceptive exposure, 18 sessions* **Comparator**: ET plus placebo (*n* = 40), supportive counselling (SC) plus naltrexone (*n* = 42), SC plus placebo (*n* = 43)	**MH**: PSS‐I **SUD**: TLFB, ACS **Measurement points**: baseline, post‐treatment, 6‐month follow up	**All interventions reduced PTSD symptom severity at post‐treatment and 6‐month follow up, no condition was superior** – *ET ± naltrexone*: baseline (M = 30.3, SD = 8.39, CI 27.7–32.9), post‐treatment (M = 12.2, SD = 12.74, CI 8.2–16.1), 6 months (M = 7.9, SD = 12.42, CI 4.1–11.8) – *ET ± placebo*: baseline (M = 27.7, SD = 9.84, CI 24.7–30.8), post‐treatment (M = 13.3, SD = 12.90, CI 9.3–17.3), 6 months (M = 10.8, SD = 14.35, CI 6.3–15.2) – *SC ± naltrexone*: baseline (M = 27.1, SD = 10.08, CI 24.7–30.8), post‐treatment (M = 15.3, SD = 10.58, CI 12.2–18.6), 6 months (M = 10.9, SD = 12.23, CI 7.2–14.6) – *SC ± placebo*: baseline (M = 27.5, SD = 10.20, CI 24.7–30.8), post‐treatment (M = 15.5, SD = 10.37, CI 12.4–18.6), 6 months (M = 11.1, SD = 9.86, CI 8.2–14.1) **Naltrexone significantly reduced % of days drinking and craving compared to placebo at post‐treatment** – *% days drinking*: naltrexone (M = 5.38, SD = 14.57, CI 2.23–8.54); placebo (M = 13.29, SD = 22.47, CI 8.45–18.12); MD = 7.93^**b**^ – *Cravings*: naltrexone (M = 6.6, SD = 26.69, SD = 6.23, CI 5.2–7.9); placebo (M = 9.7, SD = 8.83, CI 7.9–11.6); MD = 3.14^**b**^ **Significant effect of ET on % days drinking from post‐treatment to 6‐month follow up** ^**b**^, **superior to SC** (MD = 3.6, SD = 26.69, CI −2.2–9.5 [ET]; MD = 15.9, SD = 33.63, CI 8.8–23.1 [SC]) **ET and naltrexone associated with lower likelihood of relapse**
Haller et al. (2016) USA	Outpatient veterans with diagnosis of depression or dysthymia and trauma exposure (with or without PTSD diagnosis) (*n* = 123) **SUD**: Alcohol only (*n* = 51), drug only (cannabinol or stimulant, *n* = 17), alcohol and drug (*n* = 55) **Age**: 47.26 **Gender**: 88.6% M, 11.4% F	**Target**: Integrated CBT (ICBT) (Phase 1) plus Cognitive Processing Therapy‐modified (CPT‐M) (Phase 2) (*n* = 61) – *ICBT: Based on intervention used in* Lydecker et al.'s (2010) *study, 24 sessions* – *CPT‐M: identifying automatic thoughts/maladaptive cognitions caused by traumatic experience, challenging thoughts through socratic questioning, modified to incorporate substance use cognitions within framework, 12 sessions* **Comparator**: ICBT alone (Phase 1 + 2) (*n* = 62)	**MH**: PCL‐C, HDRS **SUD**: TLFB, toxicology screen **Measurement points**: baseline, post phase 1, post phase 2, 12‐month follow up	*After ICBT*: **Greater durations of abstinence at post phase 1** (M = 0.82, SD = 0.28, CI 0.77–0.86) **compared to baseline** (M = 0.43, SD = 0.27, CI 0.38–0.47)^**c**^ **Significant decreases in PTSD symptom severity from baseline** (M = 56.99, SD = 13.30, CI 54.64–59.34) **to post phase 1** (M = 50.67, SD = 15.72, CI 47.89–53.44)^**c**^ **Significant decreases in depression symptom severity from baseline** (M = 33.00, SD = 10.86, CI 31.08–34.91) **to post phase 1** (M = 30.39, SD = 12.35, CI 28.30–32.57)^**c**^ **PTSD and depression improvement not ‘clinically meaningful’** *After CPT‐M*: **Duration of abstinent periods remained similar post phase 2 for both conditions** (ICBT+CPT‐M: M = 0.81, SD = 0.28, CI 0.74–0.88; ICBT only: M = 0.79, SD = 0.29, CI 0.71–0.86) (*d* = 0.07). **Greater periods of abstinence at 12 months follow‐up compared to baseline** **CPT‐M did not reduce substance use more than ICBT alone** **Greater reductions in heavy drinking in CPT‐M condition** ^**c**^ **CPT‐M did not result in a greater reduction or maintenance of PTSD symptom improvement than those receiving ICBT** (*d* = 0.19) – *CPT‐M*: post phase 1 (M = 51.46, SD = 15.48, CI 47.57–55.34), post phase 2 (M = 49.62, SD = 14.04, CI 46.09–53.14) – *ICBT*: post phase 1 (M = 49.98, SD = 15.74, CI 46.06–53.89), post phase 2 (M = 46.69, SD = 15.74, CI 42.77–50.60)
Hunter et al. (2012) USA	Participants receiving outpatient addiction treatment diagnosed with depression (mild‐major) (*n* = 73) **SUD**: Alcohol (*n* = 14), cocaine (*n* = 17), amphetamines (*n* = 23), cannabis (*n* = 14), sedatives (*n* = 1), heroin (*n* = 1), hallucinogens (*n* = 1), polysubstance (*n* = 1), other (*n* = 1) **Age**: No mean age provided (43.84% less than 30 years) **Gender**: 52.05% M, 47.95% F	**Target**: Integrated CBT for depression and SUD (*n* = 47) – *3 modules: thoughts, activites, interactions. Provided CBT strategies for identifying and modifying maladaptive cognitions, activities and interactions which relate to mood and SUD, 18 group sessions* **Comparator**: TAU (*n* = 26)	**MH**: BDI‐II, MCS **SUD**: ASI, SIP‐ad TLFB **Measurement points**: baseline, post‐treatment, 3‐month follow up	**Both groups showed significant improvements in depressive symptoms and mental health functioning from baseline to post‐treatment** ^**a**^ (CBT: −4.17, CI −10.43–2.10 [BDI‐II], −0.79, CI −6.30–4.71 [MCS])^**2**^ **and from baseline to 3‐month follow up** ^**c**^ **in depressive symptoms** (CBT: −1.53, CI −7.77–4.71)^**2**^. **No significant difference between target and comparator group** **Higher proportion of CBT group had minimal depressive symptoms at 3‐month follow up** (76% vs. 54%) **Greater improvements in CBT condition for quantity of drinks per day** (post‐treatment: −0.46, CI −1.29–0.38; 3 months: −0.43, CI −1.29–0.38 [TLFB])^**2**^ **and negative consequences from substance use** (post‐treatment: −0.71, CI −1.72–0.30; 3 months: −0.48, CI −1.42–0.46 [SIP‐ad])^**a2**^. **No significant differences between either condition** **Significant improvement in days of problem substance use from baseline to 3‐month follow up in CBT condition only** ^**a**^
Kushner et al. (2013) USA	Participants enrolled into a residential AUD treatment programme with a comorbid anxiety disorder (*n* = 344) **SUD**: Alcohol (*n* = 344), additional drug use/dependence (*n* = 114) **Age**: 39.2 **Gender**: 60.5% M, 39.5% F	**Target**: CBT—Breaking the Drinking and Anxiety Connection (CBT‐BDAC) (*n* = 171) – *Split into 3 domains with 2 sessions in each focusing on (1) anxiety symptoms and (2) link between anxiety and alcohol use: psychoeducation, cognitive restructuring, exposure/habituation—6 sessions* **Comparator**: Progressive Muscle Relaxation Training (PMRT) (*n* = 173)	**MH**: STAI **SUD**: ASI, TLFB **Measurement points**: baseline, post‐treatment, 4‐month follow up	**Lower rates of relapse in at 4‐month follow up CBT‐BDAC group** ^**a**^ – *Any drinking (TLFB)*: 41.4% (CBT‐BDAC) vs. 54.2% (PMRT) (*d* = 0.26) – *3 consecutive days drinking (TLFB)*: 19.8% (CBT‐BDAC) VS 30.5% (PMRT) (*d* = 0.25) **At 4‐month follow up, CBT‐BDAC superior in reducing frequency and quantity of alcohol consumption** – *Quantity of drinks per month (TLFB)* ^**c**^: CBT‐BDAC baseline (M = 450.87, SD = 385.72, CI 393.05–508.68), 4 months (M = 27.03, SD = 77.05, CI 15.48–38.57); PMRT baseline (M = 399.02, SD = 330.55, CI 349.76–448.27), 4 months (M = 52.02, SD = 119.67, CI 34.18–69.85) (*d* = 0.68) – *Drinking days per month (TLFB)* ^**b**^: CBT‐BDAC baseline (M = 20.44, SD = 8.76, CI 19.12–21.75), 4 months (M = 2.03, SD = 5.59, CI 1.19–2.86); PMRT baseline (M = 20.67, SD = 9.14, 95% CI 19.30–22.03), 4 months (M = 3.30, SD = 6.85, CI 2.27–4.32), (*d* = 0.42) – *Binge days per month (TLFB)* ^**c**^: CBT‐BDAC baseline (M = 19.71, SD = 9.13, CI 18.34–21.07), 4 months (M = 1.71, SD = 4.95, CI 0.96–2.45); PMRT baseline (M = 19.51, SD = 9.56, CI 18.08–20.93), 4 months (M = 3.01, SD = 6.66, CI 2.01–4.00) (*d* = 0.48) **Large reductions in state and trait anxiety for both groups post‐treatment, treatment effect not significant** – *Trait*: CBT‐BDAC 15.17 point reduction (SD = 11.68, CI 13.41–16.92), PMRT 13.20 point reduction (SD = 9.72, CI 11.75–14.64) (*d* = 0.18) – *State*: CBT‐BDAC 12.57 point reduction (SD = 14.16, CI 10.44–14.69), PMRT 12.32 point reduction (SD = 12.63, CI 10.43–14.20) (*d* = 0.01) **Trait anxiety significantly lower in CBT‐BDAC group at 4‐month follow up** ^**a**^ (M = 41.41, SD = 12.36, CI 39.55–43.26)
Lydecker et al. (2010) USA	Veterans with diagnosis of major depressive disorder (*n* = 206) **SUD**: Alcohol only (*n* = 43), drug only (cannabinol and/or stimulant, *n* = 2), alcohol and drug (*n* = 161) **Age**: 48.2 **Gender**: 92% M, 8% F	**Target**: ICBT plus pharmacotherapy (*n* = 107) – *3 modules (1) thoughts, identifying maladaptive cognitions, thought challenging techniques; (2) identifying/scheduling activites which increase positive affect, (3) assertiveness and communication training, increasing positive interactions, refusal skills—reinforcement of these skills, 20 sessions* **Comparator**: Twelve‐Step Facilitation Therapy plus pharmacotherapy (TSF + *P*) (*n* = 99)	**MH**: HDRS **SUD**: ASI, TLFB **Measurement points**: baseline, post phase 1, post phase 2; 3‐, 6‐, 9‐ and 12‐month follow up	**Greater reductions in frequency of drug and alcohol use over time in those receiving ICBT compared to comparator at 12‐month follow up** – *% days abstinent (TLFB)*: ICBT M = 84%, CI 79–90 vs. M = 74%, CI 66–83 **Both interventions resulted in decreases in depressive symptom severity, improvements in ICBT only slightly greater however not significant** (*d* = 0.09) – *ICBT*: post‐phase 2 (M = 24.5, SD = 16.09, CI 21.4–27.5), 12 months (M = 22.5, SD = 15.83, CI 19.5–25.5) – *TSF ± P*: post‐phase 2 (M = 18.8, SD = 12.69, CI 16.3–21.3), 12 months (M = 21.1, SD = 15.22, CI 18.0–24.0)
McGovern et al. (2011) USA	Outpatients who met diagnostic criteria for PTSD (*n* = 53) **SUD**: Polydrug use (*n* = 53) **Age**: 37.85 **Gender**: 43.4% M, 56.6% F	**Target**: ICBT (*n* = 32) – *Similar to intervention's used by* Capone et al. (2018) *and* McGovern et al. (2015),*12–14 sessions* **Comparator**: Individual Addiction Counselling (IAC) (*n* = 21)	**MH**: BDI, CAPS, PCL **SUD**: ASI, TLFB, alcohol breathlyser, urine drug screen **Measurement points**: baseline, post‐treatment, 3‐month follow up	**ICBT reduced global PTSD symptom severity to a greater degree than IAC** ^**a**^ (*d* = −0.17) – *ICBT*: baseline (M = 75.75, SD = 19.94, CI 68.84–82.65), post‐treatment (M = 36.08, SD = 19.19, CI 29.43–42.72), 3 months (M = 46.50, SD = 21.75, CI 38.96–54.03) – *IAC*: baseline (M = 84.19, SD = 22.57, CI 74.53–93.84), post‐treatment (M = 52.60, SD = 21.86, CI 43.25–61.95), 3 months (M = 49.75, SD = 28.64, CI 37.50–61.99) **Greater proportion no longer had PTSD diagnosis in ICBT at 3‐month follow up** **Significant reduction in substance use severity over in both groups from baseline to 3‐month follow up** ^**c**^, **IAC superior** – *Alcohol severity (ASI)*: ICBT baseline (M = 0.25, SD = 0.29, CI −0.15–0.35), 3 months (M = 0.11, SD = 0.20, CI 0.04–0.17), MD = 0.14; IAC baseline (M = 0.24, SD = 0.22, CI 0.14–0.33), 3 months (M = 0.03, SD = 0.06, CI 0.00–0.05), MD = 0.19 (*d* = 0.75) – *Drug severity (ASI)*: ICBT baseline (M = 0.19, SD = 0.15, CI 0.3–0.24), 3 months (M = 0.10, SD = 0.09, CI 0.06–0.13), MD = 0.09; IAC (M = 0.20, SD = 0.09, CI 0.16–0.23), 3 months (M = 0.10, SD = 0.10, CI 0.05–0.14), MD = 0.10 (*d* = 0.03) **Significant reduction in alcohol and drug use days in both groups from baseline to 3 month follow up** ^**c**^ – *Alcohol (TLFB)*: 64.6% (ICBT) vs. 98.4% reduction (IAC) (*d* = 0.63) – *Drug (TLFB)*: 65.04% (ICBT) vs. 30.1% reduction (IAC) (*d* = 0.41) **Participants with more severe PTSD experienced greater improvement in PTSD symptom severity, ICBT produced greater improvement in drug use** ^**a**^ **and psychiatric symptom severity** ^**a‐c**^ **in this sub‐sample**
McGovern et al. (2015) USA	Participants receiving outpatient addiction treatment who met diagnostic criteria for PTSD (*n* = 221) **SUD**: Polydrug use, primarily mixed alcohol (*n* = 135) and opioid use (*n* = 113) **Age**: 35.3 **Gender**: 40.7% M, 59.3% F	**Target**: ICBT plus TAU (*n* = 73) – *Similar to interventions used by* Capone et al. (2018) *and* McGovern et al. (2011), *8–12 sessions* **Comparator**: IAC plus TAU (*n* = 75), TAU alone (*n* = 73)	**MH**: CAPS **SUD**: ASI‐alcohol, ASI‐drug, TLFB, urine drug screen **Measurement points**: baseline, 6 months	**PTSD declined significantly over time in all treatment conditions** ^**a**^, **no difference between all conditions** (*d* = −0.12 [ICBT vs. IAC]) – *ICBT*: baseline (M = 76.71, SD = 18.13, CI 72.55–80.86), 6 months (M = 46.81, SD = 24.81, CI 41.11–52.50) – *IAC*: baseline (M = 78.79, SD = 21.36, CI 73.95–83.62), 6 months (M = 49.62, SD = 25.71, CI 43.80–55.43) – *TAU*: baseline (M = 76.51, SD = 20.83, CI 7.73–81.28), 6 months (M = 52.60, SD = 26.46, CI 46.53–58.67) **ICBT had greater reductions in frequency of drug use than TAU** ^**a**^, **no difference between ICBT and IAC nor frequency of alcohol use in all conditions** – *Drug (TLFB)*: ICBT baseline (M = 21.27, SD = 25.09, CI 15.51–27.02), 6 months (M = 7.76, SD = 18.76, CI 3.45–12.06); IAC baseline (M = 20.13, SD = 26.16, CI 14.21–26.05), 6 months (M = 9.49, SD = 20.11, CI 4.93–14.04), TAU baseline (M = 31.64, SD = 29.15, CI 24.95–38.32), 6 months (M = 18.30, SD = 28.73, CI 11.70–24.89) (*d* = −0.09 [ICBT vs. IAC]) – *Alcohol (TLFB)*: ICBT baselines (M = 18.04, SD = 23.11, CI 12.53–23.34), 6 months (M = 4.95, SD = 12.25, CI 2.14–7.76); IAC baseline (M = 13.67, SD = 19.84, CI 9.18–18.16), 6 months (M = 3.82, SD = 11.34, CI 1.25–6.38); TAU baseline (M = 13.82, SD = 20.57, CI 9.10–18.53), 6 months (M = 4.92, SD = 16.10, CI 1.22–8.61) (*d* = 0.10 [ICBT vs. IAC]) **Significant reduction in drug toxicology results at 6‐month follow up in ICBT compared to other conditions** ^**a**^ – *Positive toxicology*: 18.5% (ICBT) vs. 37.7% (IAC) vs. 38.8% (TAU)
Mills et al. (2012) Australia	Participants who met diagnostic criteria for PTSD (*n* = 103) **SUD**: Polydrug dependence **Age**: 33.7 **Gender**: 62.1% M, 37.9% F	**Target**: COPE plus TAU (*n* = 55) **Comparator**: TAU (*n* = 48)	**MH**: CAPS **SUD**: CIDI **Measurement point**: baseline, 6 weeks, 3 months, 9 months	**COPE had significantly greater reductions in PTSD symptom severity compared to TAU from baseline to 9 months** ^**a**^ – *COPE*: MD = −38.24, CI −47.93, −28.54 – *TAU*: MD = −22.14, CI −30.33, −13.95 – *COPE* vs. *TAU*: MD = 16.09 M CI −29.00, −3.19 (*d* = −0.45) **Duration of abstinence periods and quantity of drug used did not differ between groups, both continued to use substances at 9‐month follow‐up** (81.8% [COPE] vs. 72.9% [TAU]) **Both groups showed significant reductions in severity of dependence** ^**c**^ **but no difference between groups** (0.57 [COPE] vs. 0.60 [TAU]; IRR 0.96, CI 0.69–1.34)
Morley et al. (2014) Australia	Participants with high suicide risk (i.e., recent suicide attempt, current suicidal ideation/plan) (*n* = 185) **SUD**: Alcohol (*n* = 157) and polydrug use, primarily cannabis (*n* = 110) and amphetamine (*n* = 56) **Age**: 35.61 **Gender**: 70.3% M, 29.7% F	**Target**: Opportunistic Cognitive Behavioural Package (OCB) (*n* = 122) – *CBT strategies targeting substance use and depressive symptoms/suicidal ideation, including: relapse prevention strategies, identifying cognitions linked with suicidality, developing problem solving strategies, challenging thoughts linked with depression/suicide, 8 sessions* **Comparator**: TAU (*n* = 63)	**MH**: BSS, HADS, presence of suicidal ideation/behaviour **SUD**: C‐DAEF, SES, quantity of alcohol/cannabis **Measurement point**: baseline, 6‐month follow up	**Quantity of alcohol** ^**b**^ **and cannabis** ^**c**^ **use significantly decreased in both OCB and TAU, no differences between either group (no mean outcome data provided for SUD)** **Frequency of alcohol** ^**c**^ **and cannabis** ^**c**^ **use significantly decreased in both OCB and TAU, no differences between either group** **Reduction in alcohol consumption not maintained at follow‐up** **Significant improvement in measures of depression** ^**c**^, **anxiety** ^**c**^ **and suicidal ideation** ^**c**^ **from baseline to 6‐month follow up, this was not affected by treatment group** – *BSS*: OCB baseline (M = 9.50, SD = 9.61, CI 7.79–11.20), 6 months (M = 5.82, SD = 5.58, CI 4.83–6.81); TAU baseline (M = 12.56, SD = 8.55, CI 10.44–14.67), 6 months (M = 6.00, SD = 6.61, CI 4.36–7.63) (*d* = −0.03) – *HADS (depression)*: OCB baseline (M = 9.30, SD = 6.66, CI 8.11–10.48), 6 months (M = 6.40, SD =5.43, CI 5.43–7.36); TAU baseline (M = 9.89, SD = 5.60, CI 8.50–11.27), 6 months (M = 7.10, SD = 4.30, CI 6.03–8.16) (*d* = −0.13) – *HADS (anxiety)*: OCB baseline (M = 12.58, SD = 6.66, CI 11.39–13.76); 6 months (M = 8.83, SD = 5.72, CI 7.81–9.84); TAU baseline (M = 12.35, SD = 5.39, CI 11.01–13.68), 6 months (M = 9.71, SD = 4.30, CI 8.64–10.77) (*d* = −0.16) **Marked reduction in suicide‐related outcomes (attempts/ideation) observed in both groups**
Morley et al. (2016) Australia	Participants with historical/current diagnosis of depression or anxiety disorder (*n* = 37) **SUD**: Alcohol dependence **Age**: 41.59 **Gender**: 54.1% M, 45.9% F	**Target**: Integrated Care (IC) (*n* = 21) – *3–4 period of stabilisation on naltrexone or acamprosate, followed by sessions focused on cognitive restrucruing, graded exposure, coping skills training and motivational approaches to alcohol consumpton, 7–10 sessions* **Comparator**: TAU (*n* = 16)	**MH**: DASS‐21 **SUD**: OCDS, ADS, TLFB **Measurement points**: baseline, weeks 3, 12, 16 and 24	**Participants receiving IC had significantly longer number of days until relapse at week 12** (IC: M = 46.50, SD = 10.88, CI 41.84–51.15; TAU: M = 14.20. SD = 8.38, CI 10.09–18.30)^**a**^ (*d* = 3.35) **and lapse following stabilisation period than TAU** (IC: M = 42.75, SD = 10.06, CI 38.44–47.05; TAU: M = 7.80, SD = 2.16, CI 6.74–8.85)^**c**^ (*d* = 4.64) **Significant impact of IC on number of days abstinent at week 12** (IC: M = 80.69, SD = 10.78, CI 76.07–85.30; TAU: M = 49.94, SD = 11.00, CI 44.55–55.33)^**a**^ (*d* = 2.90) **IC did not significantly improve symptoms of anxiety and depression compared to TAU**
Najavits et al. (2018) USA	Veterans who met diagnostic criteria for PTSD (*n* = 52) **SUD**: Polydrug use, most met criteria for dependence (*n* = 49), primarily alcohol (*n* = 37) **Age**: 48.75 **Gender**: 73.1% M, 26.9% M	**Target**: Creating Change (CC) (*n* = 26) – *Processing of traumatic and painful SUD memories, exploration of themes related to past experiences, 17 sessions* **Comparator**: Seeking Safety (SS) (*n* = 26)	**MH**: PTSD Checklist, TRGI **SUD**: ASI, Beliefs about Substance Use, toxicology **Measurement points**: baseline, post‐treatment, 3‐month follow up	**Significant improvement in PTSD symptoms from baseline to 3‐month follow up in both conditions** ^**b**^ (*d* = 0.24) – *CC*: baseline (M = 50.37, SD = 14.09, CI 44.95–55.78), 3 months (M = 45.85, SD = 16.67, CI 39.44–52.25) – *SS*: baseline (M = 52.75, SD = 12.41, CI 47.98–57.52), 3 months (M = 42.43, SD = 12.44, CI 37.64–47.21) **Significant improvement in alcohol** ^**c**^ **and drug use** ^**a**^ **from baseline to 3‐month follow up in both conditions** – *Alcohol (ASI)*: CC baseline (M = 0.31, SD = 0.20, CI 0.23–0.38), 3 months (M = 0.14, SD = 0.13, CI 0.09–0.19); SS baseline (M = 0.41, SD = 0.21, CI 0.32–0.49), 3 months (M = 0.19, SD = 0.12, CI 0.14–0.23) (*d* = −0.41) – *Drug (ASI)*: CC baseline (M = 0.07, SD = 0.08, CI 0.03–0.10), 3 months (M = 0.05, SD = 0.05, CI 0.03–0.06), SS baseline (M = 0.05, SD = 0.07, CI 0.02–0.07), 3 months (M = 0.04, SD = 0.06, CI 0.01–0.06) (*d* = 0.18) **Significant improvement in SUD cognitions from baseline to 3‐month follow up in both conditions** ^**a**^ (*d* = 0.00) – *CC*: baseline (M = 48.75, SD = 16.92, CI 42.2–55.2), 3 months (M = 44.78, SD = 22.28, CI 36.2–53.3) – *SS*: baseline (M = 44.73, SD = 20.67, CI 36.78–52.67), 3 months (M = 31.91, SD = 16.53; CI 25.55–38.26) **No difference between either condition on any outcome measures**
Norman et al. (2019) USA	Veterans with full or subthreshold PTSD diagnosis (*n* = 119) **SUD**: Alcohol only **Age**: 41.6 **Gender**: 89.9% M, 10.1% F	**Target**: COPE (*n* = 63) **Comparator**: SS (*n* = 56)	**MH**: CAPS **SUD**: TLFB **Measurement points**: baseline, post‐treatment, 3‐month follow up, 6‐ month follow up	**Significantly greater decease in PTSD symptom severity in those receiving COPE compared to SS** ^**b**^ (*d* = −0.44) – *COPE*: baseline (M = 43.2, SD = 12.96, CI 40.0–46.4), 6 month (M = 22.5, SD = 17.41, CI 18.2–26.8) – *SS*: baseline (M = 42.1, SD = 12.98, 95% CI 38.7–45.5), 6 month (M = 29.8, SD = 15.84, CI 25.6–33.9) **Higher rates of PTSD remission in those receiving COPE** ^**a**^ **—maintained at 3‐ and 6‐month follow up** **Improvement in frequency and quantity of alcohol use in both interventions, however no statistically significant difference between COPE or SS** – *% heavy drinking days*: COPE baseline (M = 52.5, SD = 24.5, CI 46.5–58.6), 6 months (M = 20.2, SD = 33.61, CI 11.9–28.5); SS baseline (M = 50.4, SD = 24.05, CI 44.1–56.7), 6 months (M = 19.9, SD = 29.58, CI 12.1–27.6) (*d* = 0.01) – *% days abstinent*: COPE baseline (M = 34.3, SD = 29.35, CI 27.1–41.6), 6 months (M = 66.2, SD = 39.28, CI 56.5–75.9); SS baseline (M = 31.2, SD = 29.20, CI 23.5–38.8), 6 months (M = 64.0, SD = 35.31, CI 54.8–73.3) (*d* = 0.06)
Ruglass et al. (2017) USA	Participants who met diagnostic criteria for full or subthreshold PTSD (*n* = 110) **SUD**: Polydrug and alcohol dependence, alcohol main primary substance (*n* = 49) **Age**: 44.8 **Gender**: 63.6% M, 36.4% F	**Target**: COPE (*n* = 39) **Comparator**: RP (*n* = 43), Active Monitoring Control Group (AMCG) (*n* = 28)	**MH**: CAPS, MPSS‐R **SUD**: ASI, SUI **Measurement points**: baseline, post‐treatment, 1‐, 2‐ and 3‐month follow up	**COPE and RP produced significant reductions in PTSD symptom severity (over 7**‐**day period) compared with AMCG. No difference between COPE and RPT** – *MPSS‐R*: COPE‐AMCG (−34.06, CI—51.35 to −29.68)^**c**^ (*d* = −0.92); RP‐AMCG (−22.58, CI –36.9 to −8.24)^**b**^ (*d* = −0.55) **COPE and RP produced significant reductions in PTSD symptom severity (over 30‐day period) at 1‐ and 3‐month follow up, no difference between conditions (no follow up data obtained for AMCG)** – *CAPS*: 1 month: COPE (−27.12, CI –35.84 to 18.40)^**c**^, RP (−25.38, CI –33.12 to −17.64)^**c**^ (*d* = 0.01), 3 months: COPE (−28.31, CI –36.01 to −20.60)^**c**^, RP (−26.71, CI −34.28 to −19.14)^**c**^ (*d* = −0.02) **COPE resulted in greater reduction of PTSD symptom severity in sub‐sample of full PTSD participants** ^**a**^, **no difference between COPE and RP amongst participants with subthreshold PTSD** **Both treatments showed significant reductions in past 7 days of primary substance use from baseline to post‐treatment compared with AMCG. RP superior to COPE** – *SUI*: COPE‐AMCG (−0.97, CI −1.72 to −0.22)^**c**^ (*d* = −0.51); RP‐AMCG (−2.07, CI −2.92 to −1.21)^**c**^ (*d* = −1.54); RP‐COPE (−1.10, 95% CI −2.18 TO −0.02)^**a**^ (*d* = 0.69) **COPE and RP produced significant reductions in past 30 days of primary substance use at both 1‐ and 3‐month follow up, no difference between conditions** – *ASI*: 1 month: COPE (−9.67, CI −13.65 to −5.73)^**c**^, RP (−13.40, CI −16.97 to −9.83)^**c**^ (*d* = 0.59); 3 months: COPE (−10.45, CI −14.27 to −6.63)^**c**^, RP (−13.36, CI −17.97 to −8.74)^**c**^ (*d* = 0.48)
Sannibale et al. (2013) Australia	Participants with diagnosis of PTSD (*n* = 62) **SUD**: Alcohol only, 95% alcohol dependent (*n* = 59) **Age**: 41.1 **Gender**: 47% M, 53% F	**Target**: Integrated Therapy (IT) (*n* = 33) – *Combined CBT for AUD plus cognitive restructuring and exposure therapy for PTSD, included identifying trauma‐related distortions and it's relation to drinking*, in vivo *exposure and relapse prevention, 12 sessions* **Comparator**: Alcohol Support (AS) (CBT for AUD plus supportive counselling) (*n* = 29)	**MH**: CAPS‐5, BDI‐II, STAI, PDS **SUD**: TLFB, SADQ‐C, SIP **Measurement points**: baseline, post‐treatment, 5‐ and 9‐month follow up	**Lower alcohol dependence scores (SADQ‐C) reported by AS** (M = 19.10, SD = 17.04, CI 12.89–25.30) **than IT** (M = 25.92, SD = 20.08, CI 19.96–32.77) **at 5‐month follow up** ^**b**^ (*d* = 0.37) **Lower alcohol consumption, dependence levels and alcohol related problems reported by AS than IT** – *Drinks per drinking day (TLFB)*: IT baseline (M = 13.41, SD = 7.36, CI 10.89–15.92), 5 months (M = 8.81, SD = 5.89, CI 6.80–10.82), 9 months (M = 6.97, SD = 4.16, CI 5.55–8.38); AS baseline (M = 15.99, SD = 6.89, CI13.49–18.49), 5 month (M = 6.91, SD = 6.22, CI 4.64–9.17)^**b**^, 9 months (M = 7.90, SD = 6.24, CI 5.62–10.17) (*d* = −0.18) – *% days abstinent (TLFB)*: IT baseline (median = 10, range = 0–76), 5 months (median = 35, range = 0–89), 9 months (media*n* = 27.5, range = 0–90); AS baseline (median = 13, range = 0–58), 5 months (media*n* = 32, range = 0–90)^**a**^, 9 months (media*n* = 48.5, range = 0–90) – *Alcohol related problems (SIP)*: IT baseline (M = 25.61, SD = 10.43, CI 22.05–29.16), 5 months (M = 20.46, SD = 13.25, CI 15.93–24.98), 9 months (M = 20.38, SD = 13.61, CI 15.73–25.02); AS baseline (M = 27.92, SD = 11.08, CI 23.88–31.95), 5 months (M = 14.95, SD = 13.90, CI 9.89–20.00), 9 months (M = 15.33, SD = 13.58, CI 15.33–13.58) (*d* = 0.37) **No significant between group differences on PTSD symptom reduction. Higher rates of improvement in IT compared to AS** (55% [IT] vs. 32% [AS], OR 2.53, 95% CI: 1.27–5.04) **Only IT participants who attended one or more exposure therapy session showed two fold greater likelihood of a clinically significant reduction in PTSD symptoms (55% of IT sample)**
Schäfer et al. (2019) Germany	Participants with full or subthreshold PTSD (*n* = 343) **SUD**: Polydrug and alcohol use, mainly alcohol (*n* = 293), cannabis (*n* = 165) and sedatives (*n* = 106) **Age**: 40.9 **Gender**: 100% F	**Target**: SS (*n* = 111) – *Integrated PTSD/SUD intervention, present‐focused, teaches CBT skills and interpersonal techniques, e.g., safe coping skills (e.g., asking for help), 16 sessions (2 individual, 14 group)* **Comparator**: Relapse Prevention Training (RPT) (*n* = 115), TAU (*n* = 117)	**MH**: BDI‐II, DERS, PDS, PSS‐I **SUD**: ASI‐lite **Measurement points**: baseline, post‐treatment, 3‐ and 6‐month follow up	**Significant main effect of time on both interviewer rated and self‐reported PTSD symptom severity** ^**b**^, **no significant differences between groups** – *PDS*: SS baseline (M = 25.7, SD = 11.2, CI 23.61–27.78), 6 months (M = 19.4, SD = 11.9, CI 17.18–21.61); RPT baseline (M = 27.6, SD = 10.0, CI 25.77–29.42), 6 months (M = 19.9, SD = 11.7, CI 17.76–22.03); TAU baseline (M = 27.7, SD = 10.2, CI 25.85–29.54), 6 months (M = 23.7, SD = 12.5, CI 21.43–25.96) (*d* = −0.27 [SS vs. TAU]; *d* = 0.08 [SS vs. RPT]) – *PSS‐I*: SS baseline (M = 25.4, SD = 9.7, CI 23.59–27.20), 6 months (M = 22.1, SD = 11.5, CI 19.96–24.23); RPT (M = 27.5, SD = 9.80, CI 25.79–29.29), 6 months (M = 20.7, SD = 11.0, CI 18.69–22.71); TAU baseline (M = 28.9, SD = 9.4, CI 27.19–30.60), 6 months (M = 24.3, SD = 11.4, CI 22.23–26.36) (*d* = −0.15 [SS vs. TAU]; *d* = 0.20 [SS vs. RPT]) **No significant time by group interaction effect on any substance use outcome, no significant differences between SS and either RPT or TAU. SS and RPT superior to TAU on number of substance free days** (RPT‐TAU = 3.53, CI 1.08–5.98)^**b**^ **and alcohol severity** (RPT‐TAU = −0.07, CI −0.12 to −0.02^**b**^; SS‐TAU = −0.05, CI −0.10–0.02), **but not drug severity**
Simpson et al. (2022) USA	Participants who met diagnostic criteria for PTSD (*n* = 101) **SUD**: Alcohol only **Age**: 42 **Gender**: 44% M, 56% F	**Target**: Cognitive Processing Therapy (CPT‐M) (*n* = 41) – *Similar to CPT‐M, alcohol use conceptualised as an avoidance behaviour, 12 sessions* **Comparator**: RP (*n* = 38), Assessment Only (AO) (*n* = 22); after AO participants re‐randomised (CPT‐M [*N* = 53]; RP [*N* = 48])	**MH**: CAPS‐5 **SUD**: Form‐90 **Measurement points**: baseline, post‐treatment, following re‐randomisation: pre‐treatment, post‐treatment 3‐ and 12‐month follow up	**Significant improvement in PTSD symptom severity in those receiving CPT‐M compared to AO** ^**b**^ (*d* = −0.35), **no significant difference between RP and AO or between CPT‐M and AO** (*d* = 0.34) – *CPT‐M*: baseline (M = 33.51, SD = 7.64, CI 31.17–35.84), post‐treatment (M = 17.43, SD = 11.26, CI 13.98–20.87) – *RP*: baseline (M = 34.11, SD = 8.43, CI 31.43), post‐treatment (M = 21.5, SD = 12.36, CI 17.57–25.43) – *AO*: baseline (M = 32.09, SD = 8.12, CI 28.69–35.48), post‐treatment (M = 25.90, SD = 11.32, CI 21.17–30.63) **Significant decrease in frequency of heavy drinking days in both CPT‐M** ^**a**^ (*d* = −1.50) **and RP** ^**c**^ (*d* = −0.55) **compared to AO. RP superior to CPT‐M, no significant differences between any condition on frequency of drinking** – *Drinking days in past 30 days*: CPT‐M baseline (M = 16.71, SD = 10.73, CI 13.42–19.99), post‐treatment (M = 8.72, SD = 11.36, CI 5.24–12.19); RP baseline (M = 18.61; SD = 8.72, CI 15.83–21.38), post‐treatment (M = 9.54, SD = 9.53, CI 6.51–12.57); AO baseline (M = 18.64, SD = 9.86, CI 12.37–24.90), post‐treatment (M = 15.00, SD = 11.03, CI 10.39–19.60) – *Heavy drinking days in past 30 days*: CPT‐M baseline (M = 12.78, SD = 10.81, CI 9.47–16.08), post‐treatment (M = 3.40, SD = 5.50, CI 1.71–5.08); RP baseline (M = 13.27, SD = 6.68, CI 11.14–15.39), post‐treatment (M = 2.38, SD = 3.45, CI 1.28–3.47); AO baseline (M = 14.59, SD = 10.67, CI 10.13–19.04), post‐treatment (M = 9.06, SD = 10.76, CI 4.56–13.55) **Significant improvement in PTSD symptom severity and drinking amongst AO participants (post‐treatment) once re‐randomised into active treatment condition** – *PTSD*: CPT‐M pre‐treatment (M = 32.28, SD = 9.08, CI 29.83–34.72), post‐treatment (M = 19.41, SD = 14.01, CI 15.63–23.18)^**c**^; RP pre‐treatment (M = 32.00, SD = 9.62, CI 29.27–34.72), post‐treatment (M = 20.09, SD = 11.96, CI 16.70–23.47) (*d* = −0.05) – *Drinking days in past 30 days*: CPT‐M pre‐treatment (M = 16.62, SD = 10.36, CI 13.83–19.40), post‐treatment (M = 9.35, SD = 11.61, CI 6.22–12.47)^**b**^; RP pre‐treatment (M = 17.73, SD = 9.72, CI 14.98–20.48), post‐treatment (M = 10.03, SD = 10.13, CI 7.16–12.89) (*d* = −0.06) – *Heavy drinking days in past 30 days*: CPT‐M pre‐treatment (M = 11.92, SD = 10.62), post‐treatment (M = 4.94, SD = 8.25)^**c**^; RP pre‐treatment (M = 12.68, SD = 10.21, CI 9.79–15.56), post‐treatment (M = 2.97, SD = 5.62, CI 1.38–4.56) (*d* = 0.31)
Stappenbeck et al. (2015) USA	Participants with diagnosis of PTSD (*n* = 80) **SUD**: Alcohol dependence **Age**: 44.3 **Gender**: 52% M, 48% F	**Target**: Cognitive Restructuring (CR) (*n* = 31) – *Taught links between trauma exposure and maladaptive cognitions* via *the antecedent, believes and consequences framework; use of socratic questioning to challenge beliefs, principles also applied to drinking events/episodes of cravings, 1 session plus up to 4 telephone calls* **Comparator**: Experiential Acceptance (EA) (*n* = 29), attention placebo control (*n* = 20)	**MH**: PCL‐C (adapted) **SUD**: Form‐42, quantity of drinks per day **Measurement points**: baseline, 5‐week follow up	**Decrease in drinks per day in both CR and EA (2% decrease in drinking per day 1% in EA), no difference between CR and EA** – *CR*: baseline (M = 2.7, SD = 3.5, CI 1.46–3.93), 5‐week (M = 1.8, SD = 2.5, CI 0.92–2.68)^**c**^, IRR 0.59, CI 0.47–0.73 – *EA*: baseline (M = 5.2, SD = 5.9, CI 3.05–7.34), 5‐week (M = 3.6, SD = 5.0, CI 1.78–5.42), IRR 0.83, 95% CI 0.69–1.00 **Significant increase in % of days abstinent in both CR and EA group from baseline to follow‐up** (*d* = 0.48 [CR vs. EA]) – *CR*: baseline (M = 53.0, SD = 33.2, CI 41.31–64.68), 5‐week (M = 78.3, SD = 31.7, CI 67.14–89.45)^**c**^ – *EA*: baseline (M = 37.0, SD = 35.9, CI 23.93–50.06), 5‐week (M = 60.8, SD = 43.5, CI 44.96–76.63)^**b**^ **Neither CR or EA significantly reduced PTSD symptom severity** – *CR*: baseline (M = 3.9, SD = 1.6, CI 3.33–4.46), 5‐week (M = 3.1, SD = 1.8, CI 2.46–3.73) – *EA*: baseline (M = 5.2, SD = 5.9, CI 3.31–4.48), 5‐week (M = 3.6, SD = 5.0, CI 2.44–3.75)

*Note*: a = *p* < 0.05, b = *p* < 0.01, c = *p* < 0.001.

Abbreviations: ACQ, Alcohol Cravings Questionnaire; ADS, Alcohol Dependency Scale; ASI, Addiction Severity Index; AUD, alcohol use disorder; BAI, Becks Anxiety Inventory; BDI, Becks Depression Inventory; BSS, Beck Scale for Suicide Ideation; CAPS, Clinical Administered PTSD Scale; CBT, Cognitive Behavioural Therapy; C‐DAEF, Comprehensive Drug and Alcohol Evaluation Form; CDSS, Calgary Scale for Schizophrenia; CIDI, Composite International Diagnostic Interview; DASS‐21, Depression, Anxiety and Stress Scale; DERS, Difficulties in Emotional Regulation Scale; GAF, Global Assessment of Functioning; HADS, Hospital Anxiety and Depression Scale; HDRS, Hamilton Depression Rating Scale; IES‐R, Impact of Evenet Scale‐Revised; InDUC, Inventory of Drug Use Consequences; MCS, Mental Component Summary; MH, mental health condition; MI, Motivational Interviewing; MPS, Marijuana Problems Scale; MPSS‐SR, Modified PTSD Symptom Scale Self‐Report; OCDS, Obsessive Compulsive Drinking Scale; PANSS, Positive and Negative Syndrome Scale; PCL(‐C,‐M), PTSD Checklist(‐Civilian,‐Military); PDS, Post‐traumatic Disorder Scale; PSS‐I, PTSD Symptom Scale; PTSD, post‐traumatic stress disorder; RCQ, Readiness to Change Questionnaire; SADQ‐C, Severity of Alcohol Dependence Questionnaire; SES, Self‐Efficacy Scale; SIGH‐A, Structured Interview Guide for the Hamilton Anxiety Scale; SIP(‐AD), Shortened Inventory of Problems (Modified for Alcohol and Drug Use); STAI, Spielberger State–Trait Anxiety Inventory; SUD, substance use disorder; SUI, Substance Use Inventory; TAU, treatment as usual; TLFB, Timeline Follow Back; TRGI, Trauma Related Guilt Inventory.

^1^ Cohen's *d* interpreted as 0.2 = small, 0.5 = medium, 0.8 = large. Calculated using M_1_^(target)^ − M_2_^(comparator)/^SD_pooled_ at last measurement point. IRR = incidence rate ratio; OR = odds ratio.

^2^ Treatment effect estimate.

### 4.2 Risk of Bias Assessment

The outcome of the RoB 2 is outlined in Appendices 1 and 2. In summary, nine studies were judged to have a low risk of bias for the randomisation process; an additional nine were judged to be of some concern and five were judged to be of high risk (Hunter et al. 2012; Mills et al. 2012; Najavits et al. 2018; Simpson et al. 2022; Stappenbeck et al. 2015). All studies were judged to have some concern of bias for deviation from intended intervention, due to the intervention characteristics included in this review (i.e., psychological therapy) making it impossible to blind participants or clinicians to treatment assignment. Fidelity assessments were performed to mitigate contamination from clinicians in most instances, with the exception of Kushner et al. (2013) and Morley et al. (2014, 2016). However, it is plausible to assume that in all studies, participants may have been aware that they were not assigned to the target intervention, resulting in drop‐out in order to seek alternative treatment. Twenty‐two studies were judged to be of low risk of bias for missing outcome data, whilst one was judged to be of high risk (Buckner et al. 2019). Eleven studies were judged to have a low risk of bias for measurement of the outcome; ten studies were judged to be of some concern and one study was judged to be of high risk in this domain (McGovern et al. 2011). Nineteen studies were judged to be of low risk for selection of reported result; three were judged to be of some concern and one was judged to be of high risk (McGovern et al. 2015).

### 4.3 Effect of CBT Interventions

#### 4.3.1 Substance Use and Anxiety Disorders

In Buckner et al.'s (2019) study, Integrated Cannabis and Anxiety Reduction Treatment (ICART) with Motivational Enhancement Treatment‐CBT (MET‐CBT) resulted in greater abstinence from cannabis and reductions in anxiety than MET‐CBT alone. However, low rates of abstinence were reported in both conditions, particularly at the 12‐week follow‐up. Similarly, Kushner et al. (2013) found that augmenting treatment for AUD with CBT reduced frequency and quantity of drinking and relapse rates. However, when compared to Progressive Muscle Relaxation Training, CBT showed little superiority in treating anxiety.

#### 4.3.2 Substance Use and Post‐Traumatic Stress Disorder (PTSD)

Fourteen studies focused on the treatment of co‐occurring PTSD and SUD. Most RCTs used integrated forms of CBT (ICBT) (e.g., Sannibale et al. 2013; McGovern et al. 2011, 2015; Haller et al. 2016; Capone et al. 2018), which combined disorder‐specific elements of CBT into one treatment, such as cognitive restructuring with CBT for SUD (Capone et al. 2018). McGovern et al. (2015) found PTSD symptoms reduced over time irrespective of whether participants received ICBT or Addictions Counselling; this was consistent with other studies including Capone et al. (2018) and Foa et al. (2013). Significant decreases in PTSD symptom severity were also found amongst participants receiving ICBT in Haller et al.'s (2016) study. In Sannibale et al.'s (2013) study, Integrated Therapy significantly reduced PTSD severity when compared to CBT for AUD—clinically significant reductions were dependent on the number of sessions attended. Four studies used Concurrent treatment of PTSD and Substance Use Disorders using Prolonged Exposure (COPE) (i.e., Mills et al. 2012; Ruglass et al. 2017; Back et al. 2019; Norman et al. 2019) and demonstrated sustained reductions in PTSD symptom severity, with CBT showing superiority over comparator interventions in studies by Mills et al. (2012), Norman et al. (2019) and Back et al. (2019). In contrast, Ruglass et al. (2017) found that although COPE did result in reductions in PTSD symptom severity which were clinically and statistically significant, there were no differences between COPE and Relapse Prevention Therapy.

Five studies used pre‐existing CBT interventions with elements of SUD treatment that were added to a pre‐existing protocol (termed adapted monotherapy in this review). For example, by challenging alcohol‐related cognitive distortions linked to a traumatic event during Cognitive Processing Therapy (Haller et al. 2016; Simpson et al. 2022). An overall positive treatment effect of these interventions was found on PTSD symptom severity across the majority of studies. In Coffey et al.'s (2016) study, symptoms amongst participants receiving Exposure Therapy reduced significantly when compared to those receiving healthy lifestyle sessions. This finding is inconsistent with Foa et al. (2013), who found no difference between their active comparator in reducing PTSD symptoms. Similarly, Simpson et al. (2022) found Cognitive Processing Therapy to be superior to the control condition in reducing PTSD symptoms but was unable to highlight any advantage of Cognitive Processing Therapy over Relapse Prevention Therapy. Schäfer et al. (2019) found that Seeking Safety also did not confer any advantage over Relapse Prevention Training in treating PTSD. Najavits et al. (2018) found that both Creating Change and Seeking Safety effectively reduced PTSD symptoms—with no significant difference between either intervention. In addition, Haller et al. (2016) did not find that an additional course of Cognitive Processing Therapy was superior to ICBT alone. Stappenbeck et al. (2015) also found that cognitive restructuring did not result in a significant decrease in PTSD symptoms.

Findings regarding SUD (measures included frequency of use, severity of use, periods of abstinence etc.) were also heterogeneous. McGovern et al. (2011), Ruglass et al. (2017) and Haller et al. (2016) found their interventions caused significant decreases in SUD outcomes and were superior to comparator interventions (although Ruglass et al. (2017) found no significant difference between COPE and Relapse Prevention Therapy). Schäfer et al. (2019) found their intervention to be superior to treatment as usual (TAU), but not Relapse Prevention Therapy. Najavits et al. (2018) found that Creating Change and Seeking Safety were effective in reducing drug and alcohol use and maladaptive cognitions related to SUD. Sannibale et al. (2013) found that their comparator intervention was better in reducing participants' substance use. The remaining eight studies found no differences between CBT and their comparators, although CBT interventions did result in statistically significant reductions in SUD outcomes in all of these studies. Overall, a positive effect was found for integrated CBT and adapted CBT monotherapies on measures of both PTSD and SUD.

#### 4.3.3 Substance Use and Mood Disorders

Five studies used an integrated approach which included the combination of disorder specific CBT modules for SUD and depression, or the addition of SUD content onto pre‐existing depression CBT interventions. Hunter et al. (2012) found improvements in depressive and SUD symptoms (i.e., reduced quantity of drinks per day, reduced negative consequences experienced through substance use) that marginally favoured CBT, with no significant difference between either condition on depressive symptoms. Lydecker et al. (2010) and Haller et al. (2016) also found that ICBT reduced depressive symptoms. However, in both studies, improvements were modest, with Lydecker et al. (2010) finding no significant difference between either treatment condition. ICBT also led to reduced frequency and quantity of substance use in both studies, including greater durations of abstinence and improved maintenance of substance use reduction at 12‐month follow‐up. Morley et al. (2016) found that Integrated Care increased the length of abstinent periods compared to TAU. Both of Morley et al.'s (2014, 2016) studies found no difference between CBT and TAU in improving symptoms of anxiety, depression and suicide‐related measures (for Morley et al. 2014).

#### 4.3.4 Substance Use and Psychosis

Two studies (Barrowclough et al. 2010, 2014) trialled integrated CBT and motivational interviewing; Barrowclough et al. (2010) found CBT did not result in any marked improvement in clinical outcomes related to MH (e.g., severity of psychotic symptoms, frequency of hospital admissions) or SUD (i.e., frequency of substance use) in individuals with co‐occurring schizophrenia, schizophreniform or schizoaffective disorder and alcohol and/or polydrug use. Similarly, Barrowclough et al. (2014) found CBT conferred no advantage over TAU in treating symptoms of psychosis or cannabis use in individuals experiencing first episode psychosis—with no significant effect of any treatment on any measures of MH or SUD.

## 5 Discussion

Twenty‐four studies with 3164 participants were included in this review, which evaluated the effectiveness of CBT‐based approaches in treating symptoms of both MH and SUD within the DD population. The majority of studies trialled integrated forms of CBT compared with TAU or an active comparator treatment (e.g., Relapse Prevention Therapy), whilst several others (e.g., Coffey et al. 2016; Simpson et al. 2022) trialled an adapted CBT monotherapy. Studies included samples with anxiety disorders (*n* = 2); depression (*n* = 2); mixed anxiety and depression (*n* = 2; includes Morley et al.'s (2014) study on suicidality); PTSD (*n* = 16); depression and PTSD (*n* = 1) and psychosis (*n* = 2). SUDs included alcohol use/dependence only (*n* = 6), cannabis use/dependence only (*n* = 2) and samples with mixed presentations (i.e., polysubstance use, alcohol and drug use) (*n* = 15).

### 5.1 Integrated CBT Treatment

Integrated trauma interventions demonstrated efficacy in reducing both PTSD and SUD symptom severity. However, benefits were often only marginally better than SUD‐only treatments (e.g., Relapse Prevention Therapy, Addictions Counselling)—where improvements in PTSD symptoms and SUD symptoms were widely observed. For example, in Back et al.'s (2019) study, 22% of participants receiving Relapse Prevention Therapy no longer met the diagnostic criteria for PTSD (compared with 59% in COPE). A sub‐group analysis conducted by Roberts et al. (2022) found that cumulatively, studies using COPE (i.e., Back et al. 2019; Mills et al. 2012; Ruglass et al. 2017) were not superior to SUD‐only treatments in improving MH and SUD primary outcome measures. Furthermore, studies using ICBT (i.e., McGovern et al. 2015; Capone et al. 2018) and Integrated Therapy (Sannibale et al. 2013) found no significant differences in SUD and PTSD outcome measures when compared to active controls—with the exception of McGovern et al. (2011). This could be attributed to a number of factors. Firstly, the generalisation of cognitive‐behavioural skills learnt in SUD‐only interventions that target negative emotions, thinking processes and behaviours which overlap with PTSD symptoms, in addition to increased levels of self‐efficacy derived from reduced substance use—which may result in an improvement of untargeted PTSD symptoms. Secondly, participants receiving integrated treatments were offered less SUD‐focused intervention than their SUD‐only counterparts, with integrated interventions having the same dose and duration as single disorder treatments. Consequently, participants receiving the active control in Ruglass et al. (2017) and Back et al. (2019) received double the amount of SUD‐focused intervention, suggesting that participants receiving integrated interventions may have been inadequately dosed. However, similar levels of efficacy could be viewed as an advantage of integrated treatments in that two disorders are treated within the same ‘treatment episode’, indicating that COPE could produce better SUD outcomes if delivered at a higher dose.

These findings could also be explained by difficulty in participant retention, with attrition being one of the most challenging barriers in SUD research. Rates of treatment retention were low across all studies adopting an integrated approach; only 31.8% participants achieved completer status in Capone et al.'s (2018) study (i.e., attendance to 8 ICBT sessions), whilst participants attended 50% of the COPE sessions on average in Ruglass et al.'s (2017) study. Treatment attendance is highly influenced by addiction status, with drug‐use severity, frequency of heavy drinking days and AUD severity being associated with higher dropout rates (Kline et al. 2021). This is further compounded by clinical and demographic characteristics common in the DD population, such as homelessness and frequent hospitalisation. The effects of chronic use and acute withdrawal may also lead to the exacerbation of PTSD and other MH symptoms, such as hyper arousal (Lancaster et al. 2020). Improvement in MH symptoms often precedes improvement in substance use behaviour, as substances are commonly used to self‐medicate distressing symptoms (thus negatively reinforcing substance use as an avoidant coping strategy), with recovery mostly following this trajectory (Hien et al. 2010; Kaysen et al. 2014; Szafranski et al. 2017). For this reason, untreated PTSD symptoms or a slow response to treatment will often exacerbate SUD symptoms (evidence supporting the inverse is less strong), increasing risks of relapse and treatment dropout (Zandberg et al. 2016; Roberts et al. 2022; Tripp et al. 2020). Due to intrinsic factors that influence treatment non‐attendance, CBT may not be able to fully deliver its therapeutic potential for the DD population—resulting in a stagnated or prolonged recovery. Further research is needed to fully understand factors driving attrition, with consideration of how interventions can be adapted to improve treatment retention on a case‐by‐case basis.

Multiple studies in this review also trialled integrated CBT interventions for co‐occurring anxiety, depression and SUD, with mixed results. There was limited evidence to demonstrate integrated CBT as superior to active controls in reducing measures of anxiety and depression, with only two studies showing marginally better improvements (Lydecker et al. 2010; Buckner et al. 2019). However, four studies (i.e., Lydecker et al. 2010; Buckner et al. 2019; Kushner et al. 2013; Morley et al. 2016) found integrated CBT to be superior in addressing SUD outcome measures. This contradicts evidence suggesting that CBT has a ‘sleeper effect’, whereby reductions in substance use are delayed and follow improvements in depression, due to the time it takes to successfully adopt and generalise strategies learnt in sessions (Roos et al. 2020). In fact, Kushner et al. (2013) suggested that improvements in alcohol outcomes were attributed to their intervention weakening links between anxiety and alcohol—meaning that significant reductions in anxiety were not necessarily needed to precipitate a change in substance use. This further highlights the multidirectional relationship between MH and SUD and challenges aforementioned evidence regarding the sequence of symptom improvement (i.e., MH preceding SUD).

This review did not find any evidence to support the utility of integrated CBT in treating co‐occurring psychosis and cannabis use (Barrowclough et al. 2010, 2014). Lees et al. (2021) found that CBT‐based and psychosocial treatments that are effective in CUD‐only patients (e.g., contingency management) do not provide the same outcomes for patients with co‐occurring psychosis. Hjorthøj et al. (2012) highlighted that the applicability of CBT and motivational interviewing in patients with comorbid CUD and psychosis may be hindered by cognitive deficits associated with psychosis. Furthermore, cannabis use is associated with a greater severity of positive symptoms in psychosis (Schoeler et al. 2017). This could account for Barrowclough et al.'s (2010) lack of positive findings, as they used CBT and motivational interviewing as their target intervention. Furthermore, people with psychosis often perceive positive effects from their substance use, particularly as a means to manage psychotic and affective symptoms (Gregg et al. 2009). This notion may also be reinforced by cultural beliefs and, in some cases, a lack of symptomatic improvement following cessation of cannabis use. It is also worth noting that post‐treatment cannabis abstinence rates were very low (12%) in participants receiving integrated anxiety therapy (Buckner et al. 2019)—suggesting that the interplay between MH and SUD may make cessation of cannabis uniquely challenging.

### 5.2 Adapted CBT Monotherapy Treatment

All studies in this review which utilised this approach targeted co‐occurring PTSD and substance use and focused on treatments which were behavioural‐based (i.e., Prolonged Exposure Therapy) or cognitive‐based (i.e., Cognitive Processing Therapy, Seeking Safety). Results were largely congruent with integrated CBT trials, with only small statistically significant differences present in outcome measures between active treatments which targeted PTSD and SUD either in isolation or in conjunction.

In some instances, monotherapies were superior to integrated therapies in reducing PTSD symptoms. For example, Coffey et al. (2016) and Foa et al. (2013) saw an average reduction of 65% and 56% in PTSD symptoms at follow‐up, respectively across Prolonged Exposure Therapy interventions. In comparison, COPE interventions saw reductions ranging from 42% (Mills et al. 2012) to 48% (Ruglass et al. 2017). The introduction of exposure exercises at an earlier point during prolonged exposure trials compared to Mill et al.'s (2012) COPE intervention (session 3 vs. session 6) could account for this, meaning that participants received a higher dose of exposure treatment. Moreover, participants in both prolonged exposure trials had notably higher baseline PTSD symptom severity compared to COPE trials by Ruglass et al. (2017) and Norman et al. (2019; 47% reduction in PTSD symptoms). Mills et al. (2016) found that higher baseline PTSD symptom severity was positively associated with reductions in symptom severity at follow‐up. Ruglass et al. (2017) also demonstrated a greater reduction in PTSD symptom severity amongst participants with full PTSD symptoms compared to subthreshold PTSD. This could account for why trials adopting Prolonged Exposure Therapy saw greater reductions in PTSD symptom severity. The outcome of Back et al.'s (2019) COPE study, in which participants had high baseline PTSD symptom severity, were introduced to exposure exercises at session 3 and experienced a 64% reduction in PTSD symptoms—further corroborates this.

Three studies trialled Seeking Safety as an intervention for treating co‐occurring PTSD and SUD. Seeking Safety is a present‐focused coping skills model which can be delivered in both individual and group modalities and includes 25 topics which can be selected based on the clients needs (Sherman et al. 2023). Evidence supporting Seeking Safety is mixed and has demonstrated equal efficacy to active comparators in reducing PTSD symptom severity (Schäfer et al. 2019). This finding is congruent with several other RCTs in this review, including McGovern et al. (2015) and Sannibale et al. (2013). Najavits et al. (2018) also found Seeking Safety to be similar in efficacy to Creating Change, a past‐focused intervention which explores poignant themes related to a person's past, rather than a primary focus on the trauma narrative—with both interventions resulting in significant reductions in PTSD symptom severity and drug and alcohol use. Attendance rates for both interventions were high, with 67% and 68% of participants attending all sessions respectively. These are higher than completion rates on both exposure and integrated PTSD therapies found in this review, suggesting that these interventions are better tolerated. Whilst Norman et al. (2019) found COPE to be more effective than Seeking Safety in reducing PTSD symptoms (no difference between interventions on alcohol reduction), they recommended for COPE to be offered where possible. Seeking Safety and Creating Change may be suitable alternatives for individuals with DD when exposure therapies are either not available or tolerated.

Two studies trialled Cognitive Processing Therapy (Haller et al. 2016; Simpson et al. 2022), which teaches clients to challenge and replace maladaptive cognitions about the causes of traumatic events through techniques including Socratic questioning. Simpson et al. (2022) found that Cognitive Processing Therapy demonstrated similar rates of improvement in PTSD symptoms to Relapse Prevention Therapy and two studies using COPE (i.e., Mills et al. 2012; Ruglass et al. 2017). Transferability of behavioural coping strategies taught in Relapse Prevention Therapy is likely to account for this, such as managing stress reactivity associated with cravings that may be triggered by trauma‐related cues. Improvements in substance use were similar to those of other trauma interventions in this review, in that reductions in SUD severity were significant but inferior to the active control. Interestingly, Haller et al. (2016) did not find any additive benefits of Cognitive Processing Therapy in trauma‐exposed participants who had received integrated CBT for depression. They suggested that this may be because improvements in depression and increased coping ability around both mood and substance use enabled individuals to manage trauma symptoms in a similar manner. Future research should explore the sequence of PTSD symptom change and the mechanisms associated with this, both for individuals with and without a diagnosis of PTSD. These studies not only highlight Cognitive Processing Therapy as a viable alternative to integrated treatments but also show that improvements in PTSD symptoms can be derived from treatments that do not target trauma.

Stappenbeck et al. (2015) found that cognitive restructuring, a brief intervention which identifies and challenges cognitive distortions, was not effective in reducing PTSD symptom severity. Participants only received one session and up to four follow‐up telephone calls; therefore, given the dose–response relationship evidenced by other studies in this review, it is understandable that no improvements were observed. Significant reductions in alcohol use were demonstrated and maintained at follow‐up, supporting the utility of a brief coping skills intervention during an initial phase of treatment.

Whilst this review has been unable to specify superior CBT interventions, namely due to the clinical and methodological heterogeneity of the included studies and limitations in study quality identified during the quality appraisal (Appendices 1 and 2), improvements in MH and SUD outcome measures were observed across nearly all studies. Further research is needed to determine which patients are most suited to either type of therapy, and which therapeutic approaches are most effective.

### 5.3 Implications and Recommendations for Clinical Nursing Practice

This review has highlighted that CBT is an effective treatment for co‐occurring MH and SUD, albeit at similar levels of efficacy to pre‐existing SUD treatments in many instances. This suggests the need for further development of these interventions, in combination with additional research to establish mechanisms and the sequence of symptom improvement in individuals with DD receiving CBT. This review provides preliminary evidence to support the expansion of treatment options available to individuals with DD, whereby both DA and MH services could better customise their offerings to include a variety of CBT therapies (both integrated and adapted monotherapy) to suit clients' individual needs and presentations. This would need to involve careful treatment planning that is guided by formulation, risk assessment and consideration of client preferences and motivation—as it is unlikely that these interventions will be suitable for everyone.

Given the current issues faced by the UK healthcare system in providing integrated treatment for individuals with DD, MHNs are well suited to provide CBT‐based interventions to individuals with DD across DA and MH settings. This is particularly so for interventions which are easy to disseminate, do not require extensive training to deliver, are well tolerated and brief in length (e.g., Seeking Safety and cognitive restructuring). This could help to remedy skill shortages within services and contribute to symptom stabilisation during initial stages of treatment or the maintenance of skills which have already been acquired. Moreover, by developing the capacity of nurses already working with DD to deliver CBT‐based practice, accessibility would be significantly increased for individuals who are excluded from services. It could also be rewarding for MHNs to provide effective treatments. However, there are several barriers entrenched within the nursing profession which may hinder the implementation of initiatives that promote the wider use of CBT by MHNs.

One important barrier is the perceptions, attitudes and experiences of MHNs. Research has identified that MHNs often lack knowledge and awareness of issues surrounding DD care and pathology, which may cause the formation of perceptions that are based on implicit biases shaped by societal misconceptions of addiction and media portrayal of substance use. Consequently, negative and discriminatory attitudes towards the DD population sometimes permeate into the nursing profession, including the belief that substance use is the product of informed choice and hence SUD is self‐inflicted (Merrick et al. 2022). Johansson and Wiklund‐Gustin (2016) highlighted that nurses wanted to understand the function of substance use as a means of alleviating suffering but found this difficult to contextualise when patients presented as ‘normal’ in comparison to other psychiatric patients, due to drug users making a greater effort to hide their vulnerabilities. This sometimes led to the perception of drug seeking behaviours as overtly negative, ‘manipulative’ and a ‘choice’, rather than drug use being linked with experiencing MH difficulties. It is inevitable that negative attitudes towards the DD population would serve as a barrier in providing effective, person‐centred care and may prevent nurses from seeking relevant educational opportunities (van Boekel et al. 2013).

This proposition cannot be generalised to all members of the nursing profession, as researchers have demonstrated that some MHNs understood such behaviours as the product of addiction (Howard and Holmshaw 2010; Johansson and Wiklund‐Gustin 2016). However, this did not eliminate the sense of frustration MHNs experienced in caring for DD patients, perceiving some individuals as demanding and having unrealistic expectations. Frustration may stem from a difficulty in understanding the mechanisms behind such behaviours, making it understandable that clinicians often label individuals with DD as challenging (Anandan et al. 2021). The greater use of clinical supervision would enable MHNs to reflect on these feelings, process internal conflicts between perception and understanding, and support decision making around addressing problematic substance use. Howard and Holmshaw (2010) found this to be lacking in staff support structures.

It is evident that inadequate levels of knowledge regarding DD treatment will serve to hinder treatment outcomes, with MHNs lacking confidence in delivering brief SUD interventions such as motivational interviewing, goal setting and contingency management (Pinderup 2017). Wheeler et al. (2014) highlighted that MHNs were motivated to work with individuals with DD but lacked the relevant training to be able to translate this into effective practice, as working with individuals with DD involves the use of a variety of specific therapeutic approaches given the heterogeneity of the condition. The need for greater accessibility to training opportunities and improved clinical supervision has been a constant recommendation by research looking into nursing care for DD (Merrick et al. 2022). Training has also been shown to improve pre‐existing negative attitudes towards the DD population and related therapeutic interventions (Howard and Holmshaw 2010; Jackman et al. 2020). MHNs will need to be provided with ongoing support and mentoring to conduct DD interventions effectively, as training programmes delivered in isolation are extremely unlikely to result in long‐term attitudinal and behavioural change (Christie et al. 2013). Improving training standards for nurses across both community and inpatient settings with regard to DD care will need to be a pre‐requisite to delivering effective CBT intervention.

Difficulties in MHNs' understanding of DD presentations could also lie in nursing formulation and could instead be supported by incorporating client specific information into a disorder specific CBT model (see Figure 3), in a process known as case‐conceptualisation (Kuyken et al. 2008). Wilcockson (2017), who explored the role transition from MHN to CBT therapist within Improving Access to Psychological Therapies (now called NHS Talking Therapies), found that MHNs felt the nursing profession remains dominated by a medical model, with an overreliance on using a diagnostic framework during MH assessment (e.g., mental state examination). This could provide barriers in several ways. Firstly, MHNs may find it difficult to reconcile the theory–practice gap due to a lack of congruency between CBT theory and MHN practice, leading to dissonance and avoidance of delivering CBT‐based interventions. Secondly, psychological assessment is lengthy and requires sufficient time for adequate information to be able to construct a formulation. Wilcockson (2017) found that MHNs, within both in‐patient and community settings, felt they had insufficient time to talk to patients for a long period of time and that interactions remained functional and brief. This suggests that there would be little opportunity for MHNs to carry out a CBT‐based assessment in a meaningful way. The demands of the nursing role itself would need to be considered. Many services are facing shortages in MHNs, which is resulting in demand far outstripping capacity (Cranage and Foster 2022); the addition of further work responsibility could hinder nurses' ability to perform pre‐existing clinical duties, affecting the provision of high‐quality care. Simpson (2005) highlighted that community MHNs were unable to provide effective psychosocial interventions partly due to the demands of the care coordinator role, which have become more pronounced in recent years. Support structures would need to be implemented to allow nurses to deliver CBT‐based intervention effectively, such as protected time.

It may be more feasible for MHNs to provide CBT‐based interventions with DD patients once they have already been assessed, formulated according to the CBT model and received a dose of therapy. As therapeutic gains are often maintained following longer treatment periods, MHNs, especially in community settings, would be able to facilitate a continued treatment trajectory. This could have positive implications on relapse rates and improve treatment attendance; further research would be needed to assess this treatment response relationship. Training of CBT techniques could be integrated into pre‐existing DD training sessions, particularly as nurses may be suited to reinforcing and generalising strategies which patients have already acquired (e.g., Socratic questioning, application of adaptive cognitive schemas).

However, these recommendations do rely on the assumption that individuals with DD have already received intervention. Services such as NHS Talking Therapies must become more inclusive to individuals with co‐occurring SUD; they could be delivering brief substance use interventions as they are well placed to make links between common comorbidities such as anxiety and depression with alcohol and cannabis use. This would be valuable for individuals with ‘less severe’ DD, as Watkins et al. (2011) claim that providing integrated treatment within secondary MH services may be less cost effective. NHS Talking Therapies could therefore improve cost efficiency within the UK National Health Service by providing preventative CBT intervention to individuals with mild DD. Improved collaboration and communication between primary and secondary healthcare services would then improve continuity of CBT‐based care if an individual with DD required further intervention in the future. Wider conversations would also be required around the cost effectiveness of training professionals (e.g., dual diagnosis practitioners, specialist addictions nurses, and clinical psychologists) in the delivery of longer‐term, integrated CBT interventions (e.g., COPE) across secondary MH services in the National Health Service.

### 5.4 Strengths and Limitations

This review included the search of a multitude of databases, meaning a wide breadth of papers were screened to ensure as much information as possible about the use of CBT within the DD population was included. This enabled the research question to be comprehensively addressed. The consideration of methodological and theoretical flaws amongst individual studies using the RoB ensured comparison and synthesis between studies. Data synthesis could have been further enhanced by conducting a meta‐analysis of quantitative findings to determine the overall treatment effect of CBT (i.e., by each MH condition)—although the high heterogeneity present would have made it difficult to produce any meaningful results.

The identification and screening of studies was conducted by one author. When selecting studies, implicit biases may affect the validity and replicability of the results being reported. For example, there may be slight variations in the use of the eligibility criteria. In addition, the interpretation of the RoB 2 criteria may vary from author to author as critical appraisal will still rely on personal judgement. Conducting a peer review would reduce the risk of reporting bias and increase the inter‐rater reliability. Furthermore, this review only included samples who had a diagnosed MH condition—resulting in studies which focused on individuals with a SUD only being excluded, despite the likelihood of individuals with SUD presenting with MH issues.

None of the studies in this review explored issues relating to culture or equality, diversity and inclusion. This may have resulted in the loss of valuable information that could extend the generalisation of the current findings. For example, cultural factors may affect societal values and beliefs surrounding substance use and mental health, which could affect nursing practice and a patient's predisposition to seek/engage in treatment. Furthermore, the ability to implement CBT‐based practice across DA and MH services will depend on the availability of these services and the resources required to implement these interventions (i.e., nurses, clinicians, and materials). In low‐ and middle‐income countries, the acquisition of such resources may not be possible. Consequently, it is difficult to generalise these results cross‐culturally and to other healthcare systems.

## 6 Conclusions

A synthesis of the existing literature regarding the effectiveness of CBT as a treatment of DD was conducted. Overall, both disorder‐specific and integrated forms of CBT provided promising treatment outcomes for individuals with DD. However, this review struggled to ascertain evidence supporting the use of any CBT treatment over standard SUD interventions—hindered by methodological difficulties and concerns over study quality. Future high‐quality research is needed and would benefit from making direct comparisons between different types of CBT treatment, understanding mechanisms of symptom improvement, investigating how interventions could be adapted to improve treatment retention amongst the DD population, and establishing a greater understanding about what models appeal to which patients—this would help to inform feasibility for stakeholders. Further areas of exploration include trialling the efficacy of CBT in treating other DD presentations, including co‐occurring bipolar disorder or personality disorder and SUD, which frequently come into contact with secondary MH services. It could also be advantageous to explore the integration of CBT for psychosis with CBT for SUD, considering the high levels of co‐occurrence between substance use (i.e., crack cocaine, methamphetamine and cannabis) and psychosis (Fiorentini et al. 2021).

## 7 Relevance for Clinical Practice

This review has broadened understanding of the use of CBT as a therapeutic intervention in treating symptoms of MH and SUD within the DD population. It has highlighted that a diverse range of CBT‐based treatments could be used to treat DD, allowing for greater empowerment of services and stakeholders to meet the diverse needs of this patient group. Given the funding struggles experienced by DA and MH services, training MHNs to deliver CBT intervention would provide a cost‐effective solution to the treatment gap. It would also enable a greater continuity of care for patients, whereby they are being treated by the same practitioner, within the same service—this would aid the development of therapeutic relationships and be in line with treatment guidelines which advocate for integrated DD care. However, this is contingent upon the implementation of support structures that enable MHNs to employ their newly developed skills, obtain regular clinical supervision, reflect on their practice, formulate according to the CBT model and engender attitudinal changes amongst the wider nursing community. Further research is needed to establish MHNs knowledge and use of CBT within the DD population. Most importantly, this review has provided evidence that individuals with DD can often tolerate and benefit from CBT‐based treatments, reinforcing the argument that substance use should not be a contraindication for individuals with DD accessing therapy—which in some instances may be the most suitable treatment.

## Author Contributions

Both authors conceived the study and developed research methods. D.N. conducted the literature search, independently screened all articles identified from the search and interpreted the findings. D.N. also wrote the manuscript with support from A.S. Both authors revised and contributed to the editing of the final manuscript.

## Conflicts of Interest Statement

The authors declare no conflicts of interest.

## Supporting information




**Data S1:** inm70129‐sup‐0001‐Supinfo.docx.

## Data Availability

The authors have nothing to report.

## Appendix 1 Risk of Bias 2 (RoB 2) Assessment Outcome

**Table:**

Study	Randomisation process	Deviation from intended intervention	Missing outcome data	Measurement of the outcome	Selection of the reported result	Overall bias
Back et al. (2019)	Some concerns	Some concerns	Low	Low	Low	Some concerns
Barrowclough et al. (2010)	Low	Some concerns	Low	Low	Low	Some concerns
Barrowclough et al. (2014)	Low	Some concerns	Low	Low	Low	Some concerns
Buckner et al. (2019)	Some concerns	Some concerns	High	Low	Low	High
Capone et al. (2018)	Some concerns	Some concerns	Low	Some concerns	Low	Some concerns
Coffey et al. (2016)	Low	Some concerns	Low	Low	Low	Some concerns
Foa et al. (2013)	Some concerns	Some concerns	Low	Low	Low	Some concerns
Haller et al. (2016)	Some concerns	Some concerns	Low	Some concerns	Low	Some concerns
Hunter et al. (2012)	High	Some concerns	Low	Some concerns	Low	High
Kushner et al. (2013)	Low	Some concerns	Low	Some concerns	Low	Some concerns
Lydecker et al. (2010)	Some concerns	Some concerns	Low	Some concerns	Low	Some concerns
McGovern et al. (2011)	Some concerns	Some concerns	Low	High	Low	High
McGovern et al. (2015)	Low	Some concerns	Low	Some concerns	High	High
Mills et al. (2012)	High	Some concerns	Low	Some concerns	Low	High
Morley et al. (2014)	Some concerns	Some concerns	Low	Some concerns	Low	Some concerns
Morley et al. (2016)	Some concerns	Some concerns	Low	Some concerns	Low	Some concerns
Najavits et al. (2018)	High	Some concerns	Low	Low	Low	High
Norman et al. (2019)	Low	Some concerns	Low	Low	Low	Some concerns
Ruglass et al. (2017)	Low	Some concerns	Low	Low	Low	Some concerns
Sannibale et al. (2013)	Low	Some concerns	Low	Some concerns	Low	Some concerns
Schäfer et al. (2019)	Low	Some concerns	Low	Low	Low	Some concerns
Simpson et al. (2022)	High	Some concerns	Low	Low	Low	High
Stappenbeck et al. (2015)	High	Some concerns	Low	Some concerns	Low	High

## Appendix 2 Outcome of Cochrane Risk of Bias 2 Tool

**Table:**

Domain	Risk
Randomisation process	Low (*n* = 9)
	– Barrowclough et al. (2014)—use of computer generated randomisation (Barrowclough et al. 2014; Coffey et al. 2016; Norman et al. 2019) – Randomisation procedure performed by independent statistician/service with evidence of random sequence generation (Barrowclough et al. 2010; McGovern et al. 2015; Ruglass et al. 2017; Sannibale et al. 2013; Schäfer et al. 2019) – Clear evidence of allocation concealment, for example, through use of opaque envelopes (Kushner et al. 2013; Sannibale et al. 2013)
	Some concerns (*n* = 9)
	– Unequal distribution of participants across conditions, potential to bias outcomes in favour of target intervention (McGovern et al. 2011; Back et al. 2019) – Insufficient information regarding randomisation process/allocation concealment (Buckner et al. 2019; Capone et al. 2018; Foa et al. 2013; Haller et al. 2016; Lydecker et al. 2010; Morley et al. 2014, 2016)
	High (*n* = 5)
	– Target intervention twice as big as control with no information about intended allocation ratio (Hunter et al. 2012) – Significant differences in potentially important baseline characteristics outside which may not be compatible with chance (Mills et al. 2012) – Randomisation performed by study personell with no evidence of allocation concealment (Najavits et al. 2018; Simpson et al. 2022; Stappenbeck et al. 2015)
Deviation from intended intervention	Some concerns (*n* = 23)
Missing outcome data	Low (*n* = 22)
	– Use of ITT analysis, proportion of missing outcome data and reasons for dropping out similar across all intervention groups (all studies) – Use of statistical methods to correct for drop out data (e.g., Hunter et al. 2012; Sannibale et al. 2013—regression modelling)
	High (*n* = 1)
	– No information regarding how missing outcome data was handled (Buckner et al. 2019)
Measurement of outcome	Low (*n* = 11)
	– Use of toxicology to corroborate self‐reported findings (e.g., Barrowclough et al. 2010, 2014; McGovern et al. 2011, 2015; Foa et al. 2013; Ruglass et al. 2017) – Outcome assessors blinded to treatment assignment (Back et al. 2019; Coffey et al. 2016; Najavits et al. 2018; Norman et al. 2019; Ruglass et al. 2017; Simpson et al. 2022)
	Some concerns (*n* = 11)
	– Use of self‐report measures for SUD—risk of response bias (e.g., social desirability bias) (e.g., Capone et al. 2018; Haller et al. 2016; Hunter et al. 2012; Kushner et al. 2013; Mills et al. 2012; Morley et al. 2014, 2016; Sannibale et al. 2013; Stappenbeck et al. 2015) – Unclear if outcome assessors blinded to treatment assignment (Kushner et al. 2013; Lydecker et al. 2010; McGovern et al. 2015)
	High (*n* = 1)
	– Outcome assessors not blinded to treatment assignment, possibility this may have impacted follow up assessment (McGovern et al. 2015)
Selection of reported result	Low (*n* = 22)
	– Data produced appear consistent with pre‐specified analysis plan (all studies)
	High (*n* = 1)
	– Change to analysis plan once unblinded outcome data available, may have potential to bias results in favour of hypothesis (McGovern et al. 2015)
